# Association of Genetically Enhanced Lipoprotein Lipase–Mediated Lipolysis and Low-Density Lipoprotein Cholesterol–Lowering Alleles With Risk of Coronary Disease and Type 2 Diabetes

**DOI:** 10.1001/jamacardio.2018.2866

**Published:** 2018-09-19

**Authors:** Luca A. Lotta, Isobel D. Stewart, Stephen J. Sharp, Felix R. Day, Stephen Burgess, Jian’an Luan, Nicholas Bowker, Lina Cai, Chen Li, Laura B. L. Wittemans, Nicola D. Kerrison, Kay-Tee Khaw, Mark I. McCarthy, Stephen O’Rahilly, Robert A. Scott, David B. Savage, John R. B. Perry, Claudia Langenberg, Nicholas J. Wareham

**Affiliations:** 1 MRC Epidemiology Unit, Institute of Metabolic Science, University of Cambridge, Cambridge, United Kingdom; 2 MRC Biostatistics Unit, University of Cambridge, Cambridge, United Kingdom; 3 Department of Public Health and Primary Care, University of Cambridge, Cambridge, United Kingdom; 4 Oxford Centre for Diabetes, Endocrinology, and Metabolism, University of Oxford, Oxford, United Kingdom; 5 Wellcome Centre for Human Genetics, University of Oxford, Oxford, United Kingdom; 6 NIHR Oxford Biomedical Research Centre, Churchill Hospital, Oxford, United Kingdom; 7 Metabolic Research Laboratories, Institute of Metabolic Science, University of Cambridge, Cambridge, United Kingdom

## Abstract

**Importance:**

Pharmacological enhancers of lipoprotein lipase (LPL) are in preclinical or early clinical development for cardiovascular prevention. Studying whether these agents will reduce cardiovascular events or diabetes risk when added to existing lipid-lowering drugs would require large outcome trials. Human genetics studies can help prioritize or deprioritize these resource-demanding endeavors.

**Objective:**

To investigate the independent and combined associations of genetically determined differences in LPL-mediated lipolysis and low-density lipoprotein cholesterol (LDL-C) metabolism with risk of coronary disease and diabetes.

**Design, Setting, and Participants:**

In this genetic association study, individual-level genetic data from 392 220 participants from 2 population-based cohort studies and 1 case-cohort study conducted in Europe were included. Data were collected from January 1991 to July 2018, and data were analyzed from July 2014 to July 2018.

**Exposures:**

Six conditionally independent triglyceride-lowering alleles in *LPL*, the p.Glu40Lys variant in *ANGPTL4*, rare loss-of-function variants in *ANGPTL3*, and LDL-C–lowering polymorphisms at 58 independent genomic regions, including *HMGCR*, *NPC1L1*, and *PCSK9*.

**Main Outcomes and Measures:**

Odds ratio for coronary artery disease and type 2 diabetes.

**Results:**

Of the 392 220 participants included, 211 915 (54.0%) were female, and the mean (SD) age was 57 (8) years. Triglyceride-lowering alleles in *LPL* were associated with protection from coronary disease (approximately 40% lower odds per SD of genetically lower triglycerides) and type 2 diabetes (approximately 30% lower odds) in people above or below the median of the population distribution of LDL-C–lowering alleles at 58 independent genomic regions, *HMGCR*, *NPC1L1*, or *PCSK9*. Associations with lower risk were consistent in quintiles of the distribution of LDL-C–lowering alleles and 2 × 2 factorial genetic analyses. The 40Lys variant in *ANGPTL4* was associated with protection from coronary disease and type 2 diabetes in groups with genetically higher or lower LDL-C. For a genetic difference of 0.23 SDs in LDL-C, *ANGPTL3* loss-of-function variants, which also have beneficial associations with LPL lipolysis, were associated with greater protection against coronary disease than other LDL-C–lowering genetic mechanisms (*ANGPTL3 *loss-of-function variants: odds ratio, 0.66; 95% CI, 0.52-0.83; 58 LDL-C–lowering variants: odds ratio, 0.90; 95% CI, 0.89-0.91; *P *for heterogeneity = .009).

**Conclusions and Relevance:**

Triglyceride-lowering alleles in the LPL pathway are associated with lower risk of coronary disease and type 2 diabetes independently of LDL-C–lowering genetic mechanisms. These findings provide human genetics evidence to support the development of agents that enhance LPL-mediated lipolysis for further clinical benefit in addition to LDL-C–lowering therapy.

## Key Points

**Question:**

Are genetically determined differences in lipoprotein lipase (LPL)–mediated lipolysis and low-density lipoprotein cholesterol (LDL-C)–lowering pathways independently associated with risk of coronary disease and diabetes?

**Findings:**

In this genetic association study including 392 220 people, triglyceride-lowering alleles in *LPL* or its inhibitor *ANGPTL4* were associated with lower risk of coronary artery disease and type 2 diabetes in a consistent fashion across quantiles of the population distribution of LDL-C–lowering alleles. For a given genetic difference in LDL-C, the association with lower risk of coronary disease conveyed by rare loss-of-function variants in *ANGPTL3*, which are associated with lower LDL-C levels and enhanced LPL lipolysis, was greater than that conveyed by other LDL-C–lowering genetic mechanisms.

**Meaning:**

LPL-mediated lipolysis and LDL-C–lowering mechanisms independently contribute to the risk of coronary disease and diabetes, which supports the development of LPL-enhancing agents for use in the context of LDL-C–lowering therapy.

## Teaser

This genetic association study investigates the independent and combined associations of genetically determined differences in lipoprotein lipase–mediated lipolysis and low-density lipoprotein cholesterol metabolism with risk of coronary disease and diabetes.

## Introduction

Lipoprotein lipase (LPL) is an endothelium-bound enzyme that catalyzes the rate-limiting step in the clearance of atherogenic triglyceride-rich particles.^1^ There is genetic evidence of a causal link between impaired LPL-mediated lipolysis and coronary artery disease. Gain-of-function genetic variants in *LPL*^2,3^ and loss-of-function variants in its intravascular inhibitors *ANGPTL3*,^4,5,6^ *ANGPTL4*,^2,7^ and *APOC3*^8,9^ are associated with lower triglyceride levels and lower coronary disease risk, while loss-of-function variants in *LPL*^2,3,10^ and its natural activator *APOA5*^11^ are associated with higher triglyceride levels and higher coronary risk. Impaired LPL-mediated lipolysis has also been linked to insulin resistance^12^ and a higher risk of type 2 diabetes,^12,13,14,15^ but the associations of this pathway with glucose metabolism are incompletely understood.

There is growing interest around LPL-mediated lipolysis as a target for pharmacological intervention. Several new medicines that enhance LPL-mediated clearance of triglyceride-rich lipoprotein particles by directly activating LPL^16,17^ or by inhibiting its intravascular inhibitors^6,7,18,19,20^ are in preclinical^7,16,17^ or early clinical^6,18,19,20,21^ development for cardiovascular prevention. However, it is not known whether these agents will provide further benefits in addition to low-density lipoprotein cholesterol (LDL-C)–lowering therapy, which is the mainstay of lipid-lowering therapy in cardiovascular prevention. Drugs that accelerate LPL-mediated clearance of triglyceride-rich lipoprotein particles are being developed for use in addition to statins and, possibly, other LDL-C–lowering agents. However, statins,^22^ ezetimibe,^23^ and PCSK9 inhibitors^24,25,26,27^ also reduce triglyceride-rich particles, and this could limit the clinical benefits and utility of LPL-enhancing agents when used in combination with these drugs.

Large-scale clinical trials and the investment of massive resources would be required to study the effect of each of these LPL-enhancing agents on cardiovascular outcomes in the context of LDL-C–lowering therapy. In advance of outcome trials, human genetic approaches can provide evidence of whether or not genetically determined differences in LPL-mediated lipolysis and LDL-C metabolism have independent associations with cardiometabolic disease risk, which can help prioritize or deprioritize these resource-intensive efforts.^28,29^

## Methods

### Study Design

The aims of this study were to (1) investigate associations of genetically enhanced LPL-mediated lipolysis with cardiometabolic risk factors, coronary artery disease, and type 2 diabetes (eFigure 1A in the Supplement), and (2) estimate the independent and combined associations with cardiometabolic outcomes of genetically enhanced LPL-mediated lipolysis and LDL-C–lowering genetic variants (eFigure 1B and C in the Supplement). For the first aim, we estimated associations from summary-level genetic data including up to 672 505 individuals in nonstratified analyses (eFigure 1A in the Supplement). For the second aim, we used individual-level genetic data from up to 390 470 individuals from a pool of 392 220 individuals to perform 2 × 2 factorial (eFigure 1B in the Supplement) or stratified (eFigure 1C in the Supplement) genetic analyses. We also investigated the associations of naturally occurring variation in the genes encoding LPL inhibitors with cardiometabolic outcomes.

### Participants and Studies

In nonstratified analyses (eFigure 1A in the Supplement), we used genetic association data on up to 672 505 people from the European Prospective Investigation Into Cancer and Nutrition (EPIC)–InterAct,^30^ EPIC-Norfolk,^31^ UK Biobank,^32^ and large-scale genetic consortia, including the Coronary Artery Disease Genome-Wide Replication and Meta-analysis Plus the Coronary Artery Disease Genetics Consortium (CARDIoGRAMplusC4D),^33^ Diabetes Genetics Replication and Meta-analysis (DIAGRAM) consortium,^34^ Genetic Investigation of Anthropometric Traits (GIANT) consortium,^35,36^ Meta-analyses of Glucose and Insulin-Related Traits Consortium (MAGIC),^37,38^ and Global Lipids Genetics Consortium (GLGC).^39^ In factorial and stratified analyses (eFigure 1B and C in the Supplement), we used individual-level data from up to 390 470 individuals from a pool of 392 220 individuals included in EPIC-InterAct, EPIC-Norfolk, and UK Biobank (Table). EPIC-InterAct^30^ is a case-cohort study of type 2 diabetes nested within the EPIC study.^40^ EPIC-Norfolk is a prospective cohort study of more than 20 000 individuals aged 40 to 79 years living in Norfolk county in the United Kingdom at recruitment.^31^ UK Biobank is a population-based cohort of 500 000 people aged 40 to 69 years who were recruited from 2006 to 2010 from several centers across the United Kingdom.^32^ Detailed characteristics of the participants with individual-level genotype data included in this study are presented in the Table, and details about the cohorts participating in each analysis, phenotype definitions, and data sources are in eAppendix 1 and eTable 1 in the Supplement. All studies were approved by local institutional review boards and ethics committees, and participants gave written informed consent for collection of samples and genetic analysis.

**Table.** Characteristics of Participants From the UK Biobank, EPIC-InterAct, and EPIC-Norfolk Included in This Study

Characteristic	Study			
	UK Biobank	EPIC-InterAct	EPIC-InterAct	EPIC-Norfolk
**Study characteristics**				
Group	Entire cohort	Individuals with incident type 2 diabetes	Individuals without incident type 2 diabetes	Entire cohort
Country	United Kingdom	Multiple European countries	Multiple European countries	United Kingdom
Genotyping chip	Affymetrix UK BiLEVE and UK Biobank Axiom arrays	Illumina 660w quad and Illumina CoreExome chip	Illumina 660w quad and Illumina CoreExome chip	Affymetrix UK Biobank Axiom array
Imputation panel	Haplotype Reference Consortium	Haplotype Reference Consortium	Haplotype Reference Consortium	Haplotype Reference Consortium, UK10K, and 1000 Genomes
**Participant characteristics**				
Participants, No.	352 070	9400	11 593	19 157
Age at baseline, mean (SD), y	57 (8)	55 (7)	52 (9)	59 (9)
Female sex, No. (%)	189 755 (54)	4754 (51)	7231 (62)	10 175 (53)
Current smoker, No. (%)	36 464 (10)	2733 (29)	3115 (27)	2174 (11)
BMI, mean (SD)^a^	27.4 (4.8)	29.8 (4.8)	25.8 (4.1)	26.3 (3.8)
Waist-to-hip ratio, mean (SD)	0.87 (0.09)	0.92 (0.09)	0.85 (0.09)	0.86 (0.09)
Systolic blood pressure, mean (SD), mm Hg	138 (19)	144 (20)	132 (19)	135 (18)
Diastolic blood pressure, mean (SD), mm Hg	82 (10)	87 (11)	81 (11)	83 (11)
LDL-C level, mean (SD), mg/dL	NA^b^	154.4 (38.6)	146.7 (38.6)	154.4 (38.6)
HDL-C level, mean (SD), mg/dL	NA^b^	46.3 (15.4)	57.9 (15.4)	54.1 (15.4)
Triglyceride level, median (IQR), mg/dL	NA^b^	150.4 (106.2-212.4)	97.4 (70.8-141.6)	132.7 (97.4-194.7)

Abbreviations: BMI, body mass index; EPIC, European Prospective Investigation Into Cancer and Nutrition; HDL-C, high-density lipoprotein cholesterol; IQR, interquartile range; LDL-C, low-density lipoprotein cholesterol; NA, not available; UK BiLEVE, UK Biobank Lung Exome Variant Evaluation.

SI conversion factor: To convert HDL-C and LDL-C to millimoles per liter, multiply by 0.0259; triglycerides to millimoles per liter, multiply by 0.0113.

^a^ Body mass index calculated as weight in kilograms divided by height in meters squared.

^b^ As of the submission date of this article, blood lipid concentrations are still being measured in the UK Biobank study, and results are not currently available.

### Factorial and Stratified Genetic Analyses

The similarities between the random allocation of genetic variants at conception and that of participants in a randomized trial^41^ have been used as rationale to study associations of alleles in different genes to gain insights into the likely consequences of the pharmacological modulation of the gene products in a way that simulates a factorial randomized clinical trial.^42,43^ In this study, for each participant, we calculated a weighted *LPL* genetic score and a weighted LDL-C genetic score by adding the number of triglyceride-lowering *LPL* alleles or LDL-C–lowering alleles at 58 LDL-C–associated genetic loci, weighted by their effect on the corresponding lipid levels. These genetic scores were dichotomized at the median value to naturally randomize participants into 4 groups: (1) a reference group, (2) a group with genetically lower triglyceride levels via *LPL* alleles, (3) a group with genetically lower LDL-C levels via alleles at 58 independent genetic loci, and (4) a group with both genetically lower triglyceride levels via *LPL* alleles and genetically lower LDL-C levels via the 58 genetic loci. We studied associations with lipid traits and cardiometabolic outcomes between groups using a 2 × 2 factorial design (eFigure 1B in the Supplement). Further details about this approach are in eMethods 1 in the Supplement.

In stratified analyses (eFigure 1C in the Supplement), we studied the associations of *LPL* alleles with cardiometabolic outcomes in quantiles of the population distribution of 58 LDL-C–lowering alleles or alleles at 3 genes encoding the targets of current lipid-lowering therapy, including *HMGCR* (encoding the target of statins), *NPC1L1* (ezetimibe), and *PCSK9* (PCSK9 inhibitors). We considered groups above or below the median of overall and gene-specific LDL-C–lowering genetic scores as well as quintiles of the general LDL-C–lowering genetic score.

### Selection of Genetic Variants

As a proxy for genetically enhanced LPL lipolysis, we used 6 genetic variants in the *LPL* gene previously reported to be strongly and independently associated with triglyceride levels (*P* < 5.0 × 10^−8^ for each variant in conditional analyses from the GLGC^10^) (eTable 2 in the Supplement). In factorial or stratified analyses, as instruments for genetically lower LDL-C, we used 58 genetic variants from independent genomic regions associated with LDL-C levels in up to 188 577 participants of GLGC^39^ (*P* < 5.0 × 10^−8^ for LDL-C in each region; all variants were more than 500 kb away from each other and had low linkage disequilibrium, with pairwise *R*^2^ < 0.01) (eTable 2 in the Supplement). In sensitivity analyses, we used a subset of 22 of the 58 variants that were not associated with triglyceride level in GLGC.^39^ We also considered 6 *HMGCR*,^43^ 5 *NPC1L1*,^42^ and 7 *PCSK9*^43^ genetic variants previously used by Ference et al^42,43^ as genetic proxies for statin, ezetimibe, or PCSK9 inhibitor therapy (eTable 2 in the Supplement). Quality checks of genetic data and of analyses presented in this article are described in eMethods 2 in the Supplement.

### Loss-of-Function Variants in the Inhibitors of LPL

We estimated associations with cardiometabolic outcomes of a low-frequency variant in *ANGPTL4* (p.Glu40Lys; 40Lys allele frequency, 1.9%). The 40Lys allele disrupts the inhibitory effect of ANGPTL4 on LPL in vitro^44^ and is strongly associated with lower triglyceride levels (approximately 0.27 SDs lower triglycerides per 40Lys allele; *P* = 4.2 × 10^−175^) but not with LDL-C (approximately 0.004 SDs lower LDL-C per 40Lys allele; *P* = .70) in GLGC.^14^ The variant is also associated with protection from cardiometabolic disease.^2,7,14,45^

Rare loss-of-function alleles in the LPL inhibitor *ANGPTL3* are associated with lower LDL-C and triglyceride levels,^4,5,6^ offering a unique genetic model for the combined reduction of LDL-C levels and enhancement of LPL-mediated lipolysis. Genetic studies and clinical trials show that different LDL-C–lowering mechanisms protect against coronary disease with a log-linear relationship that is observed independently of the mechanism by which this reduction is attained.^42,46,47^ If the association with lower risk of *ANGPTL3* variants is only via lower LDL-C levels, one would expect their association to be the same as that of LDL-C–lowering variants in other genes for a given genetic difference in LDL-C levels. We investigated this hypothesis by meta-analyzing and modeling data from previously published genetic studies^5,6^ about the association of rare loss-of-function variants of *ANGPTL3* with LDL-C and coronary disease risk (eAppendix 2 in the Supplement). We also attempted to estimate the associations with cardiometabolic outcomes of a rare loss-of-function variant in the *APOC3* gene captured by direct genotyping in UK Biobank, but the analysis was uninformative likely because of low statistical power (eAppendix 3 in the Supplement).

### Statistical Analysis

In nonstratified and stratified genetic analyses, associations of the 6 triglyceride-lowering genetic variants in *LPL* with outcomes were estimated using weighted generalized linear regression models that accounted for correlation between genetic variants.^48^ Estimates of the association of *LPL* alleles with triglyceride levels and of *LPL* alleles with a given outcome were used to calculate estimates of the association of genetically lower triglyceride levels via *LPL* alleles with that outcome. Correlation values were obtained from the LDlink software (eTable 3 in the Supplement).^49^ Results were scaled to represent the β coefficient or the odds ratio (OR) per SD genetically lower triglyceride levels via *LPL* alleles. Triglyceride associations are expressed in natural log–transformed and standardized units. In factorial genetic analyses (eFigure 1B in the Supplement), the associations of each group relative to the reference group were estimated using linear regression for plasma LDL-C and triglyceride levels and either logistic or Prentice-weighted Cox regression (as appropriate for the study design) for coronary artery disease and type 2 diabetes.

All analyses were adjusted for age, sex, and genetic principal components. Analyses were conducted within each study and pooled using fixed-effect inverse variance–weighted meta-analysis. Statistical analyses were performed using Stata version 14.2 (StataCorp) and R version 3.2.2 (The R Foundation for Statistical Computing). A 2-tailed *P *value less than .05 was considered statistically significant.

## Results

### Associations of *LPL* Alleles With Cardiometabolic Risk Factors and Outcomes

Triglyceride-lowering alleles in *LPL* were associated with lower risk of type 2 diabetes both in combined analyses (OR per SD of genetically lower triglycerides, 0.69; 95% CI, 0.62-0.76; *P* = 2.6 × 10^−13^) (eFigure 2 and eTable 4 in the Supplement) and individual-variant analyses (eFigure 3 and eTable 5 in the Supplement). Comparisons with estimates from multiple triglyceride-lowering genetic mechanisms^50^ showed that this association is specific to *LPL* and does not reflect a general association in a protective direction of lower triglyceride levels (eAppendix 4 and eTable 6 in the Supplement). Associations with lower coronary risk (OR per SD of genetically lower triglycerides, 0.59; 95% CI, 0.53-0.66; *P* = 1.3 × 10^−22^) (eFigures 2 and 3 and eTables 4 and 5 in the Supplement) were consistent with previous studies.^10^ Triglyceride-lowering *LPL* alleles were associated with lower fasting insulin levels, fasting plasma glucose levels, and body mass index–adjusted waist-to-hip ratio (ie, a more favorable fat distribution; β in SD of body mass index–adjusted waist-to-hip ratio per SD of genetically lower triglycerides, −0.09; 95% CI, −0.12 to −0.06; *P* = 7.9 × 10^−5^) (eFigure 2 in the Supplement), a novel association consistent with evidence of the preferential LPL-mediated lipid distribution to peripheral, rather than central, adipocytes.^51^

### Independent and Combined Associations of *LPL* Alleles and LDL-C–Lowering Alleles With Cardiometabolic Outcomes

In factorial genetic analyses, people naturally randomized to genetically lower triglycerides via *LPL* alleles had lower triglyceride levels but similar LDL-C levels compared with the reference group (eFigure 4 in the Supplement). The association with lipid levels was additive to that of LDL-C–lowering alleles (eFigure 4 in the Supplement), which were also associated with lower triglyceride levels, consistent with the observed reduction in triglyceride-rich particles in people taking statins,^22^ ezetimibe,^23^ or PCSK9 inhibitors.^24,25,26,27^

**Figure 1.**
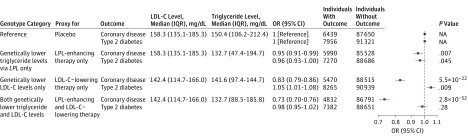
Associations of Genotype Category With Cardiometabolic Disease Outcomes in 2 × 2 Factorial Genetic Analyses Associations of each genetic score group with risk of coronary artery disease and type 2 diabetes compared with the reference group. The reference group includes those with a low-density lipoprotein cholesterol (LDL-C)–lowering score and a triglyceride-lowering *LPL* score less than or equal to the median score; the genetically lower triglyceride levels only group, those with a triglyceride-lowering *LPL *score greater than the median but an LDL-C–lowering score less than or equal to the median; the genetically lower LDL-C levels only group, those with an LDL-C–lowering score greater than the median but a triglyceride-lowering *LPL *score less than or equal to the median; and the group with both exposures, those with both scores greater than the median. Analyses include individual-level genetic data from 390 470 participants of the UK Biobank,^32^ EPIC-Norfolk,^31^ and EPIC-InterAct^30^ studies. Median values and interquartile ranges for lipid levels in a given genotype category are from the EPIC-Norfolk study. To convert LDL-C level to micromoles per liter, multiply by 0.0259. To convert triglyceride level to micromoles per liter, multiply by 0.0113. IQR indicates interquartile range; LPL, lipoprotein lipase; NA, not applicable; OR, odds ratio.

**Figure 2.**
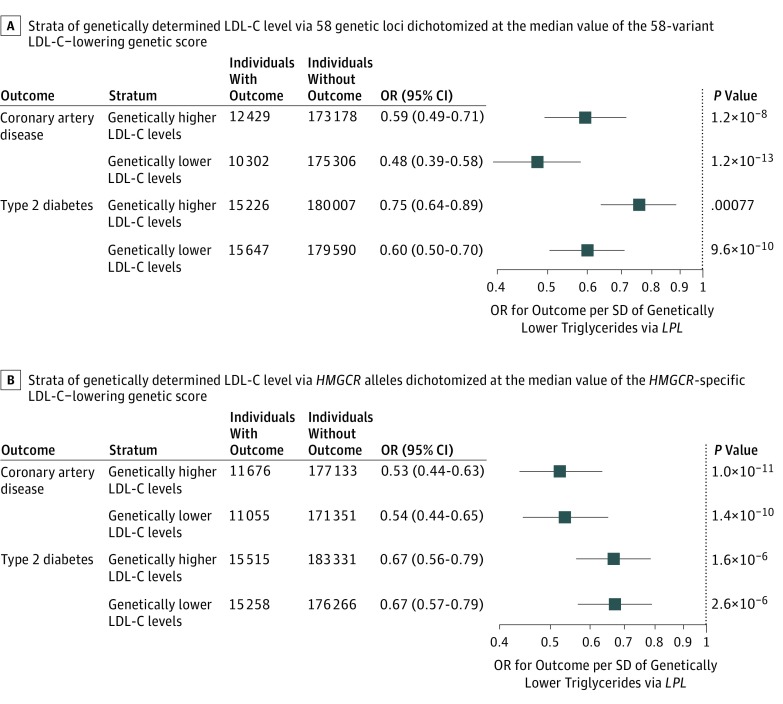
Associations of Triglyceride-Lowering *LPL* Alleles With Cardiometabolic Disease Outcomes in Individuals Above or Below the Median of the Population Distribution of Low-Density Lipoprotein Cholesterol (LDL-C)–Lowering Genetic Variants Analyses include individual-level genetic data from 390 470 participants of the UK Biobank,^32^ EPIC-Norfolk,^31^ and EPIC-InterAct^30^ studies.

**Figure 3.**
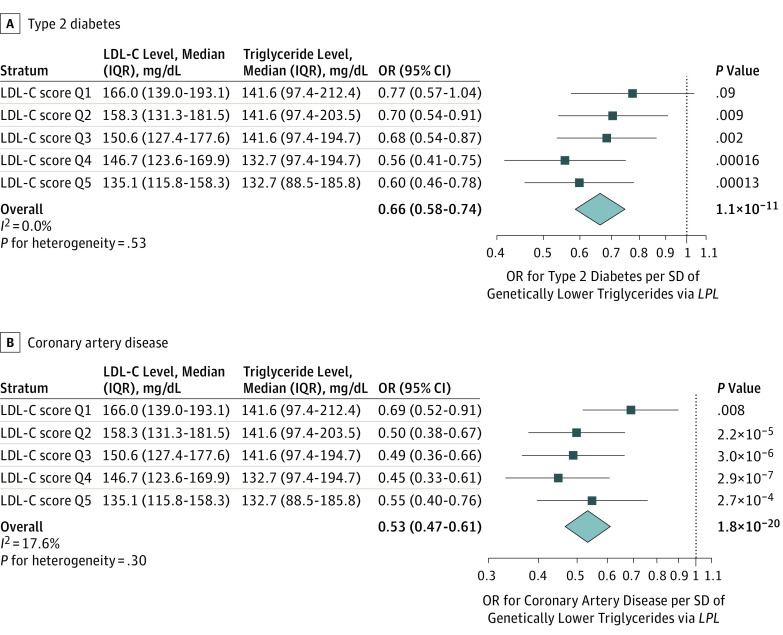
Associations of Triglyceride-Lowering *LPL* Alleles With Cardiometabolic Disease Outcomes Within Quintiles of the Population Distribution of Genetic Variants at 58 Low-Density Lipoprotein Cholesterol (LDL-C)–Associated Genetic Loci Data are from the UK Biobank,^32^ EPIC-Norfolk,^31^ and EPIC-InterAct^30^ studies. Median values and interquartile ranges for lipid levels within each stratum are from the EPIC-Norfolk study. To convert LDL-C level to micromoles per liter, multiply by 0.0259. To convert triglyceride level to micromoles per liter, multiply by 0.0113. IQR, interquartile range; OR, odds ratio.

People naturally randomized to lower LDL-C levels, lower triglyceride levels via *LPL* alleles, or both had a lower risk of coronary artery disease compared with the reference group, with the lowest odds in people naturally randomized to both genetic exposures (OR, 0.73; 95% CI, 0.70-0.76; *P* = 2.8 × 10^−52^) (Figure 1). In this group, the OR for coronary disease compared with the reference group was a further 7% (95% CI, 1%-12%) lower than expected on the basis of the association of the 2 exposures alone (*P* for interaction = .02). However, stratified analyses in groups above or below the median or in quintiles of the distribution of LDL-C–lowering alleles were not consistent with an interaction (Figure 2A and Figure 3).

People naturally randomized to lower LDL-C had a higher risk of type 2 diabetes compared with the reference group (Figure 1), consistent with previous studies.^43,50,52,53,54,55^ However, people naturally randomized to both genetic exposures had a similar risk of type 2 diabetes compared with the reference group (Figure 1), as the association of *LPL* alleles with lower risk cancelled out the risk-increasing association of LDL-C–lowering alleles. Consistently, triglyceride-lowering *LPL* alleles were strongly associated with lower diabetes risk also in people with genetically lower LDL-C levels (Figure 2A).

In stratified analyses, triglyceride-lowering *LPL* alleles were strongly and consistently associated with protection from coronary disease and diabetes in subgroups of people above or below the median of the population distribution of the 58 LDL-C–lowering alleles (Figure 2A) and of the 22 of 58 LDL-C–lowering alleles that were not associated with triglyceride levels in GLGC (eTable 7 in the Supplement), *HMGCR*, *NPC1L1*, or *PCSK9* alleles (Figure 2) (eFigure 5 in the Supplement). Associations of *LPL* alleles with lower risk were consistent in quintiles of the population distribution of the 58 LDL-C–lowering alleles (Figure 3) (eFigure 6 in the Supplement).

### Evidence From *ANGPTL4* and *ANGPTL3* Genetic Variants

**Figure 4.**
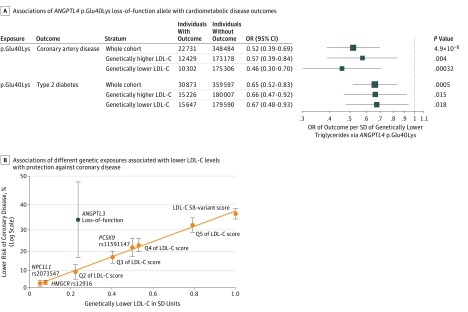
Associations of Loss-of-Function Alleles With Cardiometabolic Disease Outcomes in *ANGPTL4* and *ANGPTL3* A, Associations of the *ANGPTL4* p.Glu40Lys loss-of-function allele with cardiometabolic disease outcomes. Groups with genetically higher or lower low-density lipoprotein cholesterol (LDL-C) levels were defined on the basis of the median value of the 58-variant LDL-C–lowering genetic score. Associations are scaled to represent the odds ratio (OR) per SD of genetically lower triglyceride levels. Data are from the UK Biobank,^32^ EPIC-Norfolk,^31^ and EPIC-InterAct^30^ studies. B, Associations of different genetic exposures associated with lower LDL-C levels with protection against coronary disease. A clear log-linear relationship between genetic difference in LDL-C level and lower risk is observed for several mechanisms, while *ANGPTL3* loss-of-function variants are outliers in this relationship. For individual variants, the estimates represent per-allele differences; for quintiles of the LDL-C score, the difference is compared with the bottom quintile; for the overall genetic score, the difference is per SD of genetically lower LDL-C level; and for *ANGPTL3* variants, the difference is in carriers compared with noncarriers.

The *ANGPTL4* p.Glu40Lys variant was associated with protection from coronary disease and diabetes, with effect estimates nearly identical to the ones of triglyceride-lowering alleles in *LPL* for a given genetic difference in triglyceride levels (Figure 4A) (eFigure 2 in the Supplement). Associations were consistent in people above or below the median of the 58-variant LDL-C–lowering genetic score (Figure 4A). Also, the 40Lys allele was associated with a more favorable fat distribution in the UK Biobank (n = 350 450; SD of body mass index–adjusted waist-to-hip ratio per allele, −0.024; SE, 0.0086; *P* = .005).

In previous sequencing studies, carrying a rare loss-of-function variant in *ANGPTL3* has been associated with 36-mg/dL (to convert to millimoles per liter, multiply by 0.0113) lower triglyceride levels and 0.23-SD lower LDL-C levels (approximately 9 mg/dL).^6^ In this study, for variants at *HMGCR*, *NPC1L1*, and *PCSK9* and for the 58-variant LDL-C–lowering genetic score, a genetic difference of 0.23 SD in LDL-C was consistently associated with approximately 10% lower odds of coronary disease (OR, 0.90; 95% CI, 0.89-0.91; *I*^2^ = 0%; *P* for heterogeneity in effect estimates = .86) (eFigure 7 in the Supplement). In a meta-analysis of published genetic studies^5,6^ on rare loss-of-function variants in *ANGPTL3*, we found an association with approximately 34% lower odds of coronary disease for carriers compared with noncarriers (OR, 0.66; 95% CI, 0.52-0.83; *P* < .001; *I*^2^ = 0%; *P* for heterogeneity = .99) (eFigure 8 in the Supplement). For a given genetic difference in LDL-C level, the association of *ANGPTL3* variants with lower coronary disease risk was stronger than that of the LDL-C–lowering genetic score (*P* for heterogeneity = .009) (Figure 4B) (eFigure 7 and eTable 8 in the Supplement).

## Discussion

By analyzing individual-level genetic data in close to 400 000 people, we provide strong evidence that triglyceride-lowering alleles in the LPL pathway and LDL-C–lowering genetic mechanisms are independently associated with a lower risk of coronary artery disease. This is of relevance to the future clinical development and positioning of LPL-enhancing drugs, given that these agents are being developed for use in addition to statins and other existing LDL-C–lowering drugs. Because the LDL-C–lowering alleles studied here included those at genes encoding the targets of current LDL-C–lowering therapy, this study supports the hypothesis that pharmacologically enhancing LPL-mediated lipolysis is likely to provide further cardiovascular benefits in addition to existing LDL-C–lowering agents.

By studying the interplay of these pathways with a study design that is directly relevant to the future clinical development of LPL-enhancing agents, this study adds to previous analyses that have investigated the associations of *LPL* pathway alleles^2,3,10,12,14^ or LDL-C–lowering alleles^50,53,56,57,58^ with cardiometabolic disease separately. The independent associations with cardiometabolic outcomes of genetically enhanced LPL-mediated lipolysis and of mechanisms that lower LDL-C via *PCSK9*, *NPC1L1*, and *HMGCR* provide direct support for the development of direct enhancers of LPL^16,17^ for use in the context of existing LDL-C–lowering therapy. They also provide general support for other agents that enhance LPL activity via inhibition of its natural inhibitors in this therapeutic context.^6,7,18,19,20,21^

We also investigated variation at 2 intravascular inhibitors of LPL, *ANGPTL4* and *ANGPTL3*, making 2 important observations. First, the level of protection from coronary disease and diabetes associated with the *ANGPTL4* p.Glu40Lys variant is the same as that of *LPL* alleles for a given genetic difference in triglyceride levels and is consistent across the population distribution of LDL-C–lowering alleles. These findings are relevant for drugs that inhibit ANGPTL4^7^ or directly enhance LPL by disrupting the inhibitory activity of ANGPTL4.^17^ Second, rare loss-of-function variants in *ANGPTL3* are associated with a greater level of protection from coronary disease than other genetic mechanisms for a given genetic difference in LDL-C levels. This result suggests that ANGPTL3 inhibition may be an exception to the LDL paradigm, the mechanism-independent log-linear relationship between LDL-C lowering and coronary disease protection that has been consistently found in genetic studies and clinical trials.^42,46^ In phase 1 trials, ANGPTL3 inhibitors reduced LDL-C levels by amounts similar to or greater than currently approved LDL-C–lowering drugs.^6,20,21^ Our findings suggest that ANGPTL3 inhibitors may be more effective than current agents for a given magnitude of LDL-C reduction.

Triglyceride-lowering *LPL* alleles were also associated with protection against type 2 diabetes. The strong and consistent association of multiple independent *LPL* alleles with lower risk of type 2 diabetes found in our study extends and reinforces previous reports by us and others limited to the rs1801177^12^ and rs328^12,14,15^ alleles. We also provide evidence consistent with the association with lower odds of diabetes being specific to the LPL pathway and not being a general association of lower triglyceride levels. In factorial analyses, this association was in a protective direction with a magnitude equivalent to the association of LDL-C–lowering alleles with increased risk of type 2 diabetes. Therefore, our data suggest that enhancing LPL activity may also ameliorate glucose metabolism while further reducing the risk of cardiovascular disease in people taking LDL-C–lowering therapy.

Triglyceride-lowering alleles in *LPL* were also associated with greater insulin sensitivity, lower glucose levels, and a more favorable body fat distribution pattern, strengthening the link of this pathway with insulin and glucose metabolism.^12,45^ The novel finding from this study of robust associations of triglyceride-lowering *LPL* alleles and the *ANGPTL4* p.Glu40Lys variant with a lower waist-to-hip ratio is consistent with the known role of LPL as a lipid-buffering molecule^51^ and corroborates the notion that the association of this pathway with insulin sensitivity and lower diabetes risk may be at least partially because of improved capacity to preferentially store excess calories in peripheral adipose compartments.^12^

### Limitations

A number of assumptions and possible limitations of the genetic approach used in this study are worth considering when interpreting its results. Mendelian randomization generally assumes that genetic variants are associated with the end point exclusively via the risk factor of interest.^41^ In this case, the risk factor of interest is genetic differences in LPL-mediated lipolysis, of which triglyceride levels are a proxy, and therefore, the association of LPL alleles with different metabolic risk factors and diseases does not invalidate the approach. The consequences of modest genetically determined differences in LPL-mediated lipolysis over several decades as assessed in this study may differ from the short-term pharmacological modulation of LPL-mediated lipolysis in randomized clinical trials or clinical practice. While our analyses show a strong association of *LPL* alleles with coronary disease and diabetes, this does not necessarily mean that pharmacologically enhancing lipolysis over a short time will yield clinically relevant changes in future risk of coronary disease or new-onset diabetes in high-risk adults for whom these agents are being developed. Therefore, the effect estimates from our genetic analysis reflect a life-long exposure to genetic differences in LPL-mediated lipolysis and should not be interpreted as an exact prediction of the magnitude of the clinical effect for studies of the short-term pharmacological modulation of this pathway.

## Conclusions

Triglyceride-lowering alleles in the LPL pathway are associated with lower risk of coronary disease and type 2 diabetes independently of LDL-C–lowering genetic mechanisms. These findings provide human genetics evidence to support the development of agents that enhance LPL-mediated lipolysis for further clinical benefit in addition to LDL-C–lowering therapy.

## Notes

Supplement. **eMethods 1.** Factorial and stratified genetic analyses. **eMethods 2.** Checks of the quality of genetic data. **eAppendix 1. **Cohort descriptions and data sources. **eAppendix 2. **Associations of *ANGPTL3* loss-of-function variants with LDL cholesterol level and coronary artery disease. **eAppendix 3.** Association of a rare loss-of-function variant in *APOC3* with cardiometabolic disease outcomes in UK Biobank. **eAppendix 4.** Associations with diabetes risk of triglyceride-lowering genetic variants at the *LPL* gene or at other triglyceride-associated loci. **eFigure 1.** Design of the study. **eFigure 2.** Associations of triglyceride-lowering alleles in *LPL* with cardiometabolic risk factors and diseases. **eFigure 3.** Relationship between estimates of the association with triglyceride levels and cardiometabolic outcomes for the 6 *LPL* genetic variants. **eFigure 4.** Associations with lipid traits in 2 × 2 factorial genetic analyses. **eFigure 5.** Associations of triglyceride-lowering alleles in *LPL* with risk of coronary artery disease and type 2 diabetes in individuals above or below the median of the population distribution of genetic variants at *NPC1L1* or *PCSK9*. **eFigure 6.** Lipid levels and cardiometabolic outcomes risk in quintiles of the population distribution of genetic variants at 58 LDL-C–associated genetic loci. **eFigure 7.** Association with risk of coronary artery disease of LDL-C–lowering genetic variants at *ANGPTL3 *and other loci. **eFigure 8.** Meta-analysis of genetic association studies of *ANGPTL3* rare loss-of-function variants and risk of coronary artery disease. **eTable 1.** Data sources and participating studies. **eTable 2.** List of genetic variants in *LPL* and LDL cholesterol pathways investigated in this study. **eTable 3.** Linkage disequilibrium between *LPL* genetic variants included in the analysis. **eTable 4.** Sensitivity analysis of the association between triglyceride-lowering *LPL* alleles and risk of coronary artery disease and type 2 diabetes using only 3 variants with very low reciprocal linkage disequilibrium. **eTable 5.** Triglyceride-lowering alleles in *LPL* and risk of coronary artery disease and type 2 diabetes. **eTable 6.** Association with type 2 diabetes of triglyceride-lowering genetic variants at the *LPL* gene or at several triglyceride-associated regions studied by White et al. **eTable 7.** Sensitivity analysis of the association between triglyceride-lowering *LPL* alleles and risk of coronary artery disease and type 2 diabetes in people above or below the median of the population distribution of 22 LDL-C–lowering variants associated with LDL-C but not triglyceride levels. **eTable 8.** Heterogeneity in estimates of the association with coronary disease of *ANGPTL3* loss-of-function variants and LDL-C–lowering polygenic score in sensitivity analyses.
