# Supplementary material for: Association of Genetically Enhanced Lipoprotein Lipase–Mediated Lipolysis and Low-Density Lipoprotein Cholesterol–Lowering Alleles With Risk of Coronary Disease and Type 2 Diabetes
Source: JAMA Cardiol. 2018 Sep 19;3(10):957–66. doi: 10.1001/jamacardio.2018.2866 (PMC6217943; doi:10.1001/jamacardio.2018.2866)
Supplement: Supplement. — eMethods 1. Factorial and stratified genetic analyses. eMethods 2. Checks of the quality of genetic data. eAppendix 1. Cohort descriptions and data sources. eAppendix 2. Associations of ANGPTL3 loss-of-function variants with LDL cholesterol level and coronary artery disease. eAppendix 3. Association of a rare loss-of-function variant in APOC3 with cardiometabolic disease outcomes in UK Biobank. eAppendix 4. Associations with diabetes risk of triglyceride-lowering genetic variants at the LPL gene or at other triglyceride-associated loci. eFigure 1. Design of the study. eFigure 2. Associations of triglyceride-lowering alleles in LPL with cardiometabolic risk factors and diseases. eFigure 3. Relationship between estimates of the association with triglyceride levels and cardiometabolic outcomes for the 6 LPL genetic variants. eFigure 4. Associations with lipid traits in 2 × 2 factorial genetic analyses. eFigure 5. Associations of triglyceride-lowering alleles in LPL with risk of coronary artery disease and type 2 diabetes in individuals above or below the median of the population distribution of genetic variants at NPC1L1 or PCSK9. eFigure 6. Lipid levels and cardiometabolic outcomes risk in quintiles of the population distribution of genetic variants at 58 LDL-C–associated genetic loci. eFigure 7. Association with risk of coronary artery disease of LDL-C–lowering genetic variants at ANGPTL3 and other loci. eFigure 8. Meta-analysis of genetic association studies of ANGPTL3 rare loss-of-function variants and risk of coronary artery disease. eTable 1. Data sources and participating studies. eTable 2. List of genetic variants in LPL and LDL cholesterol pathways investigated in this study. eTable 3. Linkage disequilibrium between LPL genetic variants included in the analysis. eTable 4. Sensitivity analysis of the association between triglyceride-lowering LPL alleles and risk of coronary artery disease and type 2 diabetes using only 3 variants with very low reciprocal linkag [file jamacardiol-3-957-s001.pdf]

Supplementary Online Content

Lotta LA, Stewart ID, Sharp SJ, et al. Association of genetically enhanced lipoprotein lipase–mediated lipolysis and low-density lipoprotein cholesterol–lowering alleles with risk of coronary disease and type 2 diabetes. *JAMA Cardiol*. Published online September 19, 2018. doi:10.1001/jamacardio.2018.2866

eMethods 1. Factorial and stratified genetic analyses
eMethods 2. Checks of the quality of genetic data
eAppendix 1. Cohort descriptions and data sources
eAppendix 2. Associations of *ANGPTL3* loss-of-function variants with LDL cholesterol level and coronary artery disease
eAppendix 3. Association of a rare loss-of-function variant in *APOC3* with cardiometabolic disease outcomes in UK Biobank
eAppendix 4. Associations with diabetes risk of triglyceride-lowering genetic variants at the *LPL* gene or at other triglyceride-associated loci
eFigure 1. Design of the study
eFigure 2. Associations of triglyceride-lowering alleles in *LPL* with cardiometabolic risk factors and diseases
eFigure 3. Relationship between estimates of the association with triglyceride levels and cardiometabolic outcomes for the 6 *LPL* genetic variants
eFigure 4. Associations with lipid traits in 2 × 2 factorial genetic analyses
eFigure 5. Associations of triglyceride-lowering alleles in *LPL* with risk of coronary artery disease and type 2 diabetes in individuals above or below the median of the population distribution of genetic variants at *NPC1L1* or *PCSK9*
eFigure 6. Lipid levels and cardiometabolic outcomes risk in quintiles of the population distribution of genetic variants at 58 LDL-C–associated genetic loci
eFigure 7. Association with risk of coronary artery disease of LDL-C–lowering genetic variants at *ANGPTL3* and other loci
eFigure 8. Meta-analysis of genetic association studies of *ANGPTL3* rare loss-of-function variants and risk of coronary artery disease
eTable 1. Data sources and participating studies
eTable 2. List of genetic variants in *LPL* and LDL cholesterol pathways investigated in this study
eTable 3. Linkage disequilibrium between *LPL* genetic variants included in the analysis
eTable 4. Sensitivity analysis of the association between triglyceride-lowering *LPL* alleles and risk of coronary artery disease and type 2 diabetes using only 3 variants with very low reciprocal linkage disequilibrium
eTable 5. Triglyceride-lowering alleles in *LPL* and risk of coronary artery disease and type 2 diabetes
eTable 6. Association with type 2 diabetes of triglyceride-lowering genetic variants at the *LPL* gene or at several triglyceride-associated regions studied by White et al
eTable 7. Sensitivity analysis of the association between triglyceride-lowering *LPL* alleles and risk of coronary artery disease and type 2 diabetes in people above or below the median of the population distribution of 22 LDL-C–lowering variants associated with LDL-C but not triglyceride levels
eTable 8. Heterogeneity in estimates of the association with coronary disease of *ANGPTL3* loss-of-function variants and LDL-C–lowering polygenic score in sensitivity analyses
eReferences.

This supplementary material has been provided by the authors to give readers additional information about their work.

© 2018 Lotta LA et al. *JAMA Cardiol.*

eMethods 1. Factorial and stratified genetic analyses

Factorial genetic analysis

Factorial genetic analyses (**eFigure 1B**) were conducted in each of the UK Biobank, EPIC-InterAct or EPIC-Norfolk studies separately and then results were combined using inverse variance-weighted fixed-effect meta-analysis.

We constructed two independent *LPL* and LDL-C weighted genetic risk scores with two distinct goals: (1) to overcome the weak individual associations of genetic variants with lipid levels and disease risk and (2) to "naturally-randomize" participants into approximately equally sized groups, which ensures the greatest statistical power for these individual-level analyses and is akin to a factorial randomized controlled trial design.

We constructed these genetic scores to estimate the combined and independent association of triglyceride-lowering *LPL*-alleles and of LDL-C lowering polymorphisms at 58 genetic loci with (a) circulating lipid levels and (b) the risk of coronary artery disease and type 2 diabetes (**eFigure 1B**). We selected six genetic variants for inclusion in the *LPL* genetic score that were previously reported to be independently and strongly associated with triglyceride levels in analyses of the Global Lipids Genetics Consortium.[17] All genetic variants satisfied these criteria: (1) were in the *LPL* gene or within 10 kb of the gene; (2) were independently and strongly associated with triglyceride levels in conditional analyses of the Global Lipids Genetics Consortium with $p < 5 \times 10^{-8}$. In parallel, we built a LDL-C lowering genetic risk score using 58 genetic variants at 58 independent genetic loci reported by the Global Lipids Genetics Consortium[12] to be strongly and independently associated with LDL-C levels. All genetic variants satisfied these criteria: (1) were over 500 kb away from each other and had no or negligible linkage disequilibrium ($R^2 < 0.01$); (2) the genetic regions were associated with LDL-C levels ($p < 5 \times 10^{-8}$) in the Global Lipids Genetics Consortium analysis of up to 188,577 individuals.

For each participant and each genetic variant, we weighted the number of effect alleles (i.e. the triglyceride-lowering allele for *LPL* variants or the LDL-C lowering allele for the 58 LDL-C associated variants) for the effect on the respective lipid trait expressed in standardised units. We then dichotomised each score by dividing people in a group below or equal to the median and above the median value of the weighted score. Because polymorphisms included in genetic scores are inherited approximately randomly at the time of conception in a process known as "Mendelian randomisation",[18] and inherited approximately independently of the other polymorphisms included in the genetic score, the number of lipid lowering alleles that a person inherits for each genetic score should also be random. Therefore, partitioning the population into two groups should "naturally randomise" the population into two approximately equal groups with different genetically-determined lipid levels.

The dichotomised *LPL* and LDL-C genetic risk scores were used to naturally randomise participants into 4 groups: (1) reference, (2) genetically-lower triglycerides via *LPL*-alleles, (3) genetically-lower LDL-C via alleles at 58 independent genetic loci, or (4) *both* genetically-lower triglycerides via *LPL*-alleles *and* genetically-lower LDL-C via the 58 genetic loci (referred to as the group "naturally-randomised to both genetic exposures" for simplicity). The reference group included people below or equal to the median of both lipid-lowering genetic scores. The group "genetically-lower triglycerides via *LPL*-alleles" included

people above the median for the triglyceride-lowering *LPL* score, but below or equal to the median for the LDL-C lowering score. The group "genetically-lower LDL-C" included people below or equal to the median for the triglyceride-lowering *LPL* score, but above the median for the LDL-C lowering score. The group "naturally-randomised to both genetic exposures" included people above the median for both scores.

Using the four "naturally randomised" groups constructed as described above, the effects of each group relative to the reference group were estimated using linear regression for LDL-C and triglyceride levels, while the association with coronary artery disease and type 2 diabetes was estimated using logistic regression (for combined prevalent and incident outcomes, i.e. in UK Biobank and EPIC-Norfolk) or Cox proportional hazards models (for incident events, i.e. in the EPIC-InterAct study). All analyses were adjusted for age, sex and the first four genetic principal components.

Stratified genetic analyses

In stratified genetic analyses (**eFigure 1C**), we investigated the association of *LPL*-genetic variants with type 2 diabetes and coronary artery disease in strata of the population distribution of LDL-C lowering genetic variants. These included variants at *HMGCR* (encoding the target of statins), *NPC1L1* (encoding the target of ezetimibe) and *PCSK9* (encoding the target of PCSK9 inhibitors), the 58-variant genetic score and the 22-variant genetic score (after excluding variants associated with triglyceride levels). For each of these genes, we used sets of previously published LDL-C lowering genetic variants which were shown by Ference et al. to be strongly associated with lower LDL-C levels and lower coronary disease risk in previous genetic analyses.[19,20] We used six approximately independent genetic variants at the *HMGCR* locus, five approximately independent genetic variants at the *NPC1L1* locus and seven approximately independent genetic variants at the *PCSK9* locus.[19,20] We used these genetic variants to partition the population in two groups below or above the median of LDL-C lowering alleles (weighted for their association with LDL-C) at each locus or at the 58 or 22 loci. Additional analyses were conducted in quintiles of the 58-variant LDL-C lowering genetic score. People above the median (or in higher quinitiles) can be thought of as a group of individuals naturally randomised to lower LDL-C levels due to genetic variants at *HMGCR*, *NPC1L1* or *PCKS9* or the 58 loci, respectively, serving as a proxy for treatment with the corresponding LDL-C lowering drug or general reduction of LDL-C levels via multiple mechanisms. Within each of these resulting groups, we then estimated the associations of the six triglyceride lowering alleles at *LPL* with type 2 diabetes and coronary artery disease. We combined individual *LPL* genetic variant estimates using a weighted generalised linear regression method that accounts for the correlation between genetic variants.[21]

© 2018 Lotta LA et al. *JAMA Cardiol.*

eMethods 2. Checks of the quality of genetic data

A number of quality control procedures were used to ensure the quality of genetic data and genetic analyses presented here.

In UK Biobank, EPIC-Norfolk and the Illumina Core-Exome-genotyped subset of EPIC-InterAct, the six *LPL* genetic were directly genotyped with high-quality using genome-wide genotyping arrays. In the Illumina 660w quad genotyped subset of EPIC-InterAct, rs10096633 was directly genotyped and the other five genetic variants were imputed with minimum imputation accuracy info score of 0.91 (with a score of 1 indicating direct genotyping or perfect imputation). Genotyping in these studies underwent a number of quality control procedures including (a) routine quality checks carried out during the process of sample retrieval, DNA extraction, and genotype calling; (b) checks for genotype batch effects, plate effects, departures from Hardy-Weinberg equilibrium, sex effects, array effects, and discordance across control replicates; (c) individual and genetic variant call rate filters.

Given that UK Biobank was the largest study included in the analysis and that genetic data on close to 500,000 individuals have been recently released, we performed additional checks of the quality of data in addition to those implemented by the UK Biobank team (described in details by Bycroft and colleagues[7]). Firstly, 58 out of 82 genetic variants included in the analysis were directly genotyped in UK Biobank and cleared all pre-release quality control filters[7] as well as a further filter for >95% call rate. The remaining genetic variants were all imputed using the Haplotype Reference Consortium. Among the 89 genetic variants, the median imputation accuracy info score was 1 (with a score of 1 indicating direct genotyping or perfect imputation) and the median among 24 imputed genetic variants was 1 (minimum score 0.93), indicating excellent imputation. Because the 58 directly-genotyped genetic variants were also imputed using the Haplotype Reference Consortium, we compared the minor allele frequency in the genotyped and imputed data, finding near identical frequencies (correlation coefficient = 1.00).

For all genetic variants included in the analysis, we automatically aligned the effect allele to be coded as the lipid lowering allele (**eTable 2**), using automated scripts. The frequency of the coded effect allele was near identical in the UK Biobank, EPIC-Norfolk and EPIC-InterAct studies (correlation coefficients > 0.9987 for each pairwise comparison) and corresponded to what reported in external reference data.

In the meta-analysis of EPIC-Norfolk, EPIC-InterAct and UK Biobank results used for factorial analyses, we observed a high degree of consistency of estimates from the different studies (see **Inset Table**).

© 2018 Lotta LA et al. *JAMA Cardiol.*

Inset Table. Consistency of estimates of associations of genetic exposures with lipid traits and cardio-metabolic outcomes in factorial genetic analyses presented in this study. The I^2 and p-value for heterogeneity were used to estimate possible heterogeneity.

Genetic exposure	Outcome	Studies	Pooled Central Estimate[a]	I^2	$p_{heterogeneity}$
Lower triglycerides via-*LPL*	Triglycerides	EPIC-Norfolk, EPIC-Interact subcohort	-0.17	0%	0.51
Lower LDL-C via-58 loci			-0.12	0%	0.99
Lower triglycerides via-*LPL and* Lower LDL-C via-58 loci			-0.25	0%	0.42
Lower triglycerides via-*LPL*	LDL-C		-0.02	0%	0.68
Lower LDL-C via-58 loci			-0.46	21%	0.26
Lower triglycerides via-*LPL and* Lower LDL-C via-58 loci			-0.47	0%	0.70
Lower triglycerides via-*LPL*	Coronary artery disease	EPIC-Norfolk, UK Biobank	0.95	0%	0.80
Lower LDL-C via-58 loci			0.83	11%	0.29
Lower triglycerides via-*LPL and* Lower LDL-C via-58 loci			0.73	0%	0.94
Lower triglycerides via-*LPL*	Type 2 diabetes	EPIC-InterAct, EPIC-Norfolk, UK Biobank	0.96	16%	0.30
Lower LDL-C via-58 loci			1.05	51%	0.13
Lower triglycerides via-*LPL and* Lower LDL-C via-58 loci			0.98	52%	0.12

Abbreviations: LPL, lipoprotein lipase; LDL-C, low-density lipoprotein cholesterol; CI, confidence interval.

a Beta coefficient in standardised unit of outcome for continuous traits or odds ratio for binary outcomes compared to the reference group (i.e. people below or equal to the median of both a triglyceride lowering via *LPL* genetic score and a LDL-C lowering via 58 loci genetic score).

© 2018 Lotta LA et al. *JAMA Cardiol.*

eAppendix 1. Cohort descriptions and data sources

EPIC-InterAct

EPIC-InterAct[1] is a case-cohort study of incident type 2 diabetes nested within the European Prospective Investigation into Cancer and Nutrition (EPIC) study,[2] a cohort study of ~500,000 European participants followed-up for an average of 8 years. Eight out of the ten EPIC cohorts agreed to take part in EPIC-InterAct leaving 455,680 participants for screening. Individuals were excluded from EPIC-InterAct if they did not have stored blood (n=109,625) or information on diabetes status (n=5,821; 1.3% of participants screened for inclusion). From the remaining 340,234 participants, 12,403 individuals who developed type 2 diabetes during follow-up constituted the incident case group of EPIC-InterAct and a random group of 16,154 individuals free of diabetes at baseline constituted the subcohort group of EPIC-InterAct.[1] Incident type 2 diabetes was defined on the basis of self-report, linkage to primary care registers, secondary care registers, medication use (drug registers), hospital admissions and mortality data. Subcohort participants were previously shown to be representative of eligible EPIC participants within each country.[1] Data on a total of 20,993 participants with available genotyping (with no overlap with DIAGRAM or EPIC-Norfolk) were included in the study. Type 2 diabetes status was available in all participants. Individuals without genotype data were excluded from the study. Participant characteristics are summarised in **Table 1**.

UK Biobank

UK Biobank is a population-based cohort of over 500,000 people aged between 40-69 years who were recruited in 2006-2010 from several centres across the United Kingdom.[3] Data from UK Biobank contributed to the analyses of the associations with cardio-metabolic risk factors, type 2 diabetes and coronary artery disease. Waist and hip circumference were measured from participants using a Seca 200cm tape measure, height was measured using a Seca 240cm measure, while weight for the measurement of body mass index (BMI) was collected using a Tanita BC418MA body composition analyser. Type 2 diabetes was defined on the basis of self-reported physician diagnosis at nurse interview or digital questionnaire, age at diagnosis > 36 years, use of oral anti-diabetic medications and electronic health records.[4] Coronary artery disease was defined as either myocardial infarction or coronary disease documented in the participant's medical history at the time of enrolment by a trained nurse or hospitalisation or death involving acute myocardial infarction or its complications (i.e. International Statistical Classification of Diseases and Related Health Problems codes I21, I22 or I23), similar to what previously described.[5,6] Participant characteristics[3] and genotyping methods[7] have been reported in detail elsewhere. We describe the details of the quality checks of genetic data in **eNote 3**. Participant characteristics are summarised in **Table 1**.

EPIC-Norfolk cohort study

EPIC-Norfolk is a prospective cohort study of over 20,000 individuals aged between 40 and 79 and living in the Norfolk county in the United Kingdom at recruitment.[8] EPIC-Norfolk is a constituent cohort of the European Prospective Investigation of Cancer (EPIC).[2] Data from EPIC-Norfolk contributed to factorial and stratified genetic analyses. Coronary artery disease

was defined as either self-reported myocardial infarction at baseline or incident ischemic heart disease defined by International Statistical Classification of Diseases and Related Health Problems codes 410-414 (ICD9), or I20-I25 (ICD10). Diabetes was defined as either self-reported diabetes at baseline or incident diabetes defined by codes 250 (ICD9), or E10-E14 (ICD10). Participant characteristics and genotyping methods have been previously reported[9] and are summarised in **Table 1**.

DIAbetes Genetics Replication And Meta-analysis (DIAGRAM) Consortium
Data on type 2 diabetes has been contributed by the DIAGRAM[10] investigators and have been downloaded from: http://diagram-consortium.org/

CARDIoGRAMplusC4D Consortium
Data on coronary artery disease have been contributed by CARDIoGRAMplusC4D[11] investigators and have been downloaded from: http://www.cardiogramplusc4d.org/

Global Lipid Genetic Consortium (GLGC)
Data on LDL cholesterol, HDL cholesterol and triglycerides have been contributed by Global Lipids Genetics Consortium[12] investigators and have been downloaded from: www.sph.umich.edu/csg/abecasis/public/lipids2013/

The Genetic Investigation of ANthropometric Traits (GIANT) consortium
Data on anthropomorphic traits from the GIANT consortium[13,14] and have been downloaded from: www.broadinstitute.org/collaboration/giant/index.php/GIANT_consortium_data_files

Meta-Analyses of Glucose and Insulin-related traits Consortium (MAGIC)
Data on glycaemic traits have been contributed by MAGIC investigators[15,16] and have been downloaded from: www.magicinvestigators.org

© 2018 Lotta LA et al. *JAMA Cardiol.*

eAppendix 2. Associations of *ANGPTL3* loss-of-function variants with LDL cholesterol level and coronary artery disease

Rare loss-of-function alleles in the LPL-inhibitor *ANGPTL3* are associated with lower LDL-C and triglyceride levels,[22-24] offering a unique genetic model for the combined reduction of LDL-C levels and enhancement of LPL-mediated lipolysis. Genetic studies and clinical trials show that different LDL-C-lowering mechanisms protect against coronary disease with a mechanism-independent log-linear relationship (i.e. the “LDL-C paradigm”).[19,25,26] If the protective effect of *ANGPTL3* variants is only via LDL-C reduction, one would expect their association to be the same as that of LDL-C lowering variants in other genes, for a given genetic difference in LDL-C levels. We investigated this hypothesis by meta-analyzing and modelling data from previously published genetic studies about the association of rare loss-of-function variants of *ANGPTL3* with LDL-C and coronary disease risk.[23,24] First, we used results from Dewey and colleagues as estimates of the association of rare loss-of-function variants in *ANGPTL3* with LDL-C, i.e. 0.23 SD lower LDL-C (~0.23 mmol/L or 9 mg/dL).[23] Second, we estimated the association with coronary artery disease, for a 0.23 SD genetically-lower LDL-C, of LDL-C lowering variants at *HMGCR*, *NPC1L1*, *PCSK9* or the 58 LDL-C associated loci using data from UK Biobank and CARDIoGRAMplusC4D. Third, we estimated the association with coronary artery disease, for a 0.23 SD genetically-lower LDL-C, of rare loss-of-function variants in *ANGPTL3* by meta-analyzing genetic association studies including up to 58,399 cases and 305,796 controls (**eFigure 8**).[23,24] Fourth, we tested for heterogeneity between the estimate of the 58 LDL-C lowering alleles and that of *ANGPTL3* variants, showing evidence of heterogeneity (**eFigure 7**). We conducted a number of sensitivity analyses using different estimates for the LDL-C lowering alleles and *ANGPTL3* variants (**eTable 8**). For comparison, we show the consistency of estimates for variants at *HMGCR*, *NPC1L1*, *PCSK9* with those for the 58 variant LDL-C score (**eFigure 7**).

© 2018 Lotta LA et al. *JAMA Cardiol.*

eAppendix 3. Association of a rare loss-of-function variant in *APOC3* with cardiometabolic disease outcomes in UK Biobank

Drugs that inhibit APOC3, an inhibitor of LPL-mediated lipolysis, are in early clinical development for the treatment of dyslipidemia.[27,28] Rare loss-of-function variants in the encoding gene have been used as genetic model to study the likely consequences of pharmacological APOC3 inhibition.[29,30] These rare variants are imperfectly captured by array genotyping, such that only one of the four variants driving the reported associations was captured by direct genotyping in UK Biobank (rs147210663, p.Ala43Thr, an experimentally-validated loss-of-function variant[31]), but was not available in InterAct or EPIC-Norfolk. Nonetheless, we sought to estimate the associations with cardio-metabolic disease outcomes of this variant in UK Biobank. In 351,285 people with available genotypes, 279 carried the variant (carrier frequency 0.08%). While the carriers had lower risk of type 2 diabetes (odds ratio per copy of the rare variant rs147210663-A allele, 0.78; 95% confidence interval, 0.44-1.36; p=0.38) and coronary disease (odds ratio per copy of the rs147210663-A allele, 0.90; 95% confidence interval, 0.52-1.55; p=0.70) compared to non-carriers, the difference was not statistically significant. Therefore, it was not possible to meaningfully estimate the association of rare loss-of-function variants of *APOC3* in strata of the population distribution of LDL-C lowering alleles. Large-scale sequencing studies of the *APOC3* gene will be required to estimate this association.

© 2018 Lotta LA et al. *JAMA Cardiol.*

eAppendix 4. Associations with diabetes risk of triglyceride-lowering genetic variants at the *LPL* gene or at other triglyceride-associated loci

Studies investigating the genetic relationship between triglyceride levels and risk of type 2 diabetes have yielded conflicting results.[32-35] In a comprehensive Mendelian randomization study, White et al.[35] have estimated the genetic association between triglyceride and diabetes, using 140 triglyceride-lowering genetic variants at multiple loci accounting for possible pleiotropic effects by using different methods, including univariate, multivariate and Egger-MR Mendelian randomization analyses. They found inconsistent results between methods, with Egger-MR (a method that is robust to directional pleiotropy) estimates being consistent with a risk-increasing association for triglyceride-lowering alleles, while the two other methods showed no associations.[35] In this study, we observed associations in a protective direction between triglyceride-lowering alleles at *LPL* and diabetes risk. We asked whether this association was consistent with estimates of the general genetic relationship between triglycerides and diabetes and tested for heterogeneity between our estimates and those from White and colleagues (**eTable 6**). We found evidence of heterogeneity, suggesting that the protective association at *LPL* is specific to this gene/pathway.

eFigure 1. Design of the study

Panel A shows the design of non-stratified genetic analyses using summary-level genetic data from up to 672,505 individuals. ***Panel B*** shows the design of 2 x 2 factorial genetic analyses using individual-level genetic data from 390,470 participants of the UK Biobank, EPIC-Norfolk and EPIC-InterAct studies. ***Panel C*** shows the design of stratified genetic analyses using individual-level genetic data from 390,470 participants of the UK Biobank, EPIC-Norfolk and EPIC-InterAct studies. See eTable 1 for details about participating studies in each analysis. Abbreviations: LPL, lipoprotein lipase; LDL-C, low-density lipoprotein cholesterol; HMGCR, 3-Hydroxy-3-Methylglutaryl-CoA Reductase; NPC1L1, Niemann-Pick C1-Like 1; PCSK9, Proprotein convertase subtilisin/kexin type 9.

A

Association of triglyceride-lowering *LPL* alleles in non-stratified analyses using summary-level genetic data from up to 672,505 people

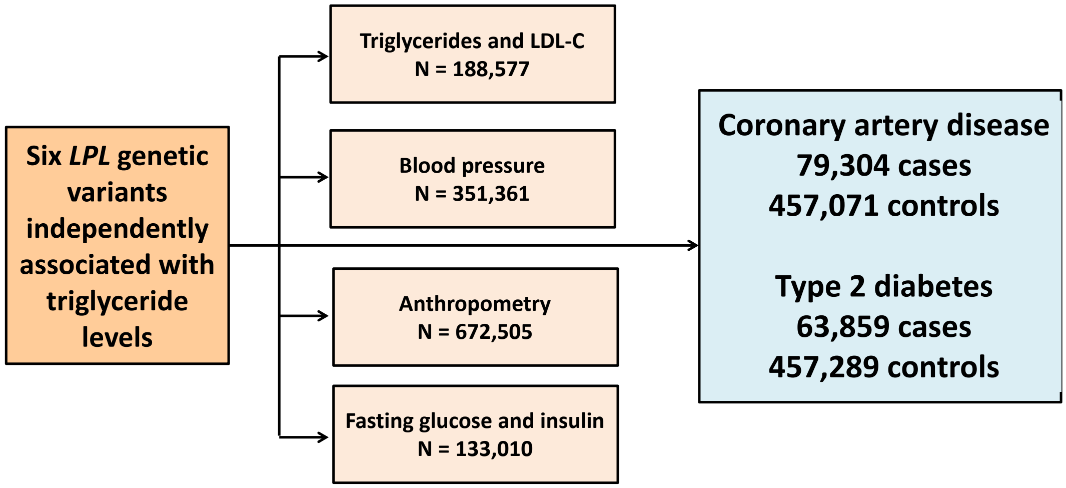

© 2018 Lotta LA et al. *JAMA Cardiol.*

B

2 x 2 factorial genetic analysis of *LPL*-alleles and LDL-C-lowering alleles using individual-level genetic data from 390,470 people

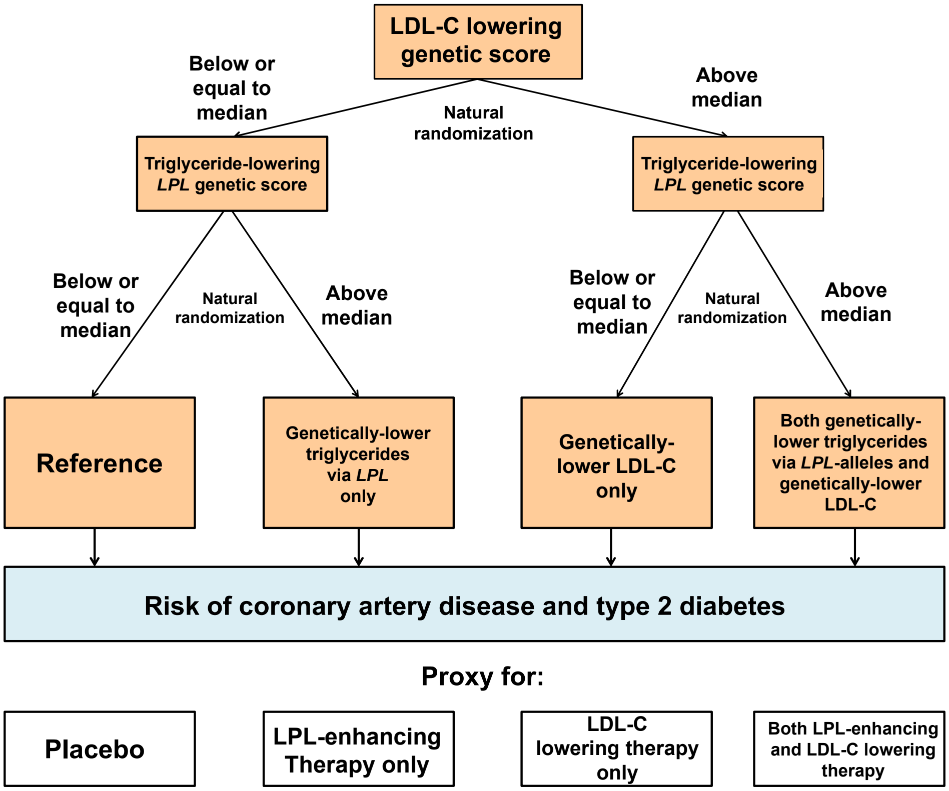

C

Association of triglyceride-lowering *LPL* alleles in stratified analyses using individual-level genetic data from 390,470 people

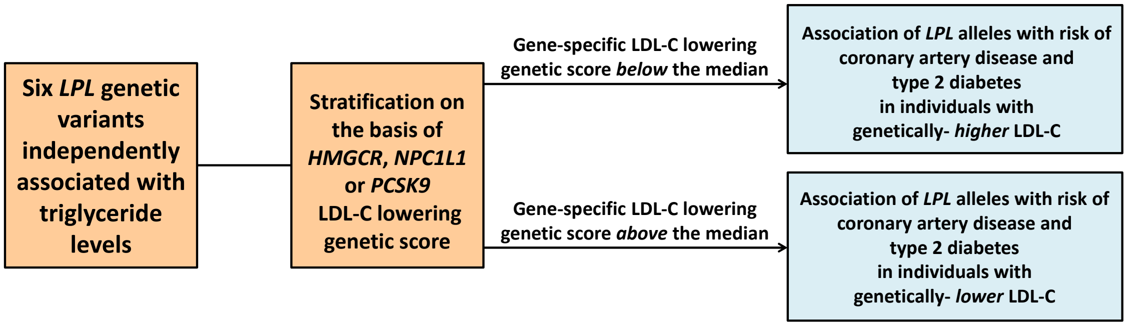

© 2018 Lotta LA et al. *JAMA Cardiol.*

eFigure 2. Associations of triglyceride-lowering alleles in *LPL* with cardiometabolic risk factors and diseases

Analyses included summary-level genetic data from up to 672,505 individuals from multiple studies (see eTable 1). The ***top panel*** shows associations with cardio-metabolic risk factors in standardized units. The ***bottom panel*** shows associations with risk of coronary artery disease and type 2 diabetes. Abbreviations: N, number of participants; CI, confidence interval; LDL-C, low-density lipoprotein cholesterol; BMI, body mass index; WHR, waist-to-hip ratio; SD, standard deviation; LPL, lipoprotein lipase; OR, odds ratio.

Phenotype	N	Beta (95% CI)	p-value
Triglycerides	188,577	-1.00 (-1.05, -0.95)	$< 10^{-300}$
LDL-C	188,577	-0.06 (-0.11, -0.00)	0.042
BMI	672,505	-0.03 (-0.06, -0.01)	0.016
WHR adjusted for BMI	559,817	-0.09 (-0.12, -0.06)	2.2×10^{-11}
Systolic blood pressure	351,354	-0.03 (-0.06, 0.00)	0.072
Diastolic blood pressure	351,361	-0.02 (-0.05, 0.01)	0.16
Fasting insulin	108,557	-0.13 (-0.19, -0.06)	7.9×10^{-05}
Fasting plasma glucose	133,010	-0.08 (-0.13, -0.03)	0.0025

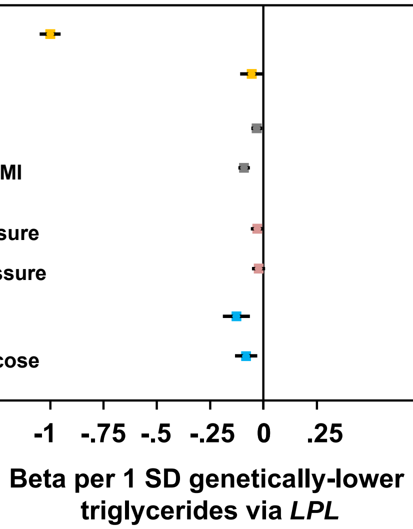

Outcome	Cases	Controls	OR (95% CI)	p-value
Coronary artery disease	79,304	457,071	0.59 (0.53, 0.66)	1.3×10^{-22}
Type 2 diabetes	63,859	457,289	0.69 (0.62, 0.76)	2.6×10^{-13}

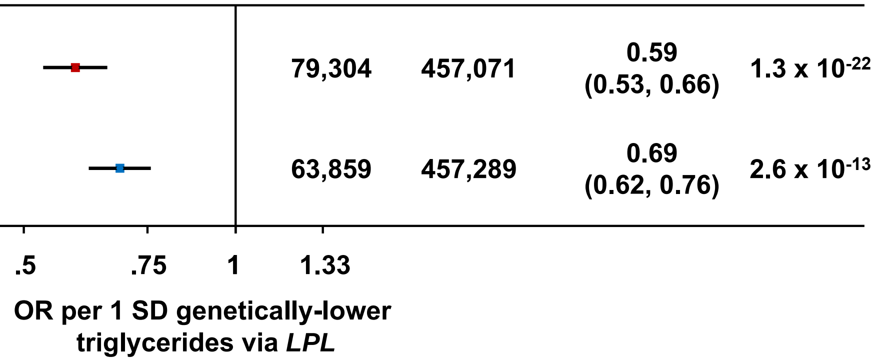

© 2018 Lotta LA et al. *JAMA Cardiol.*

eFigure 3. Relationship between estimates of the association with triglyceride levels and cardiometabolic outcomes for the 6 *LPL* genetic variants

Abbreviations: SD, standard deviation.

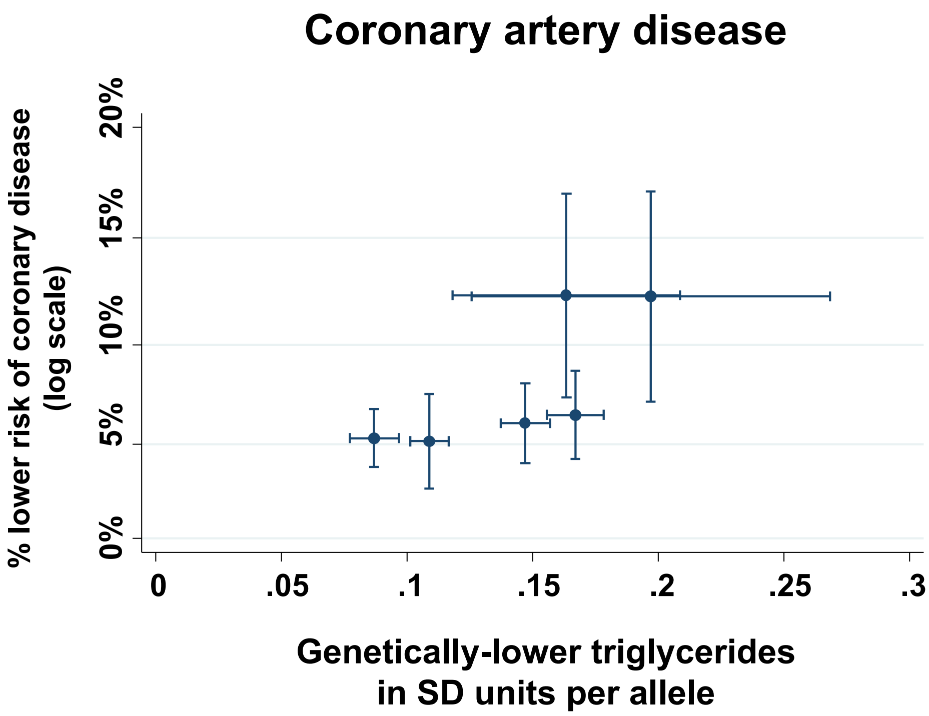

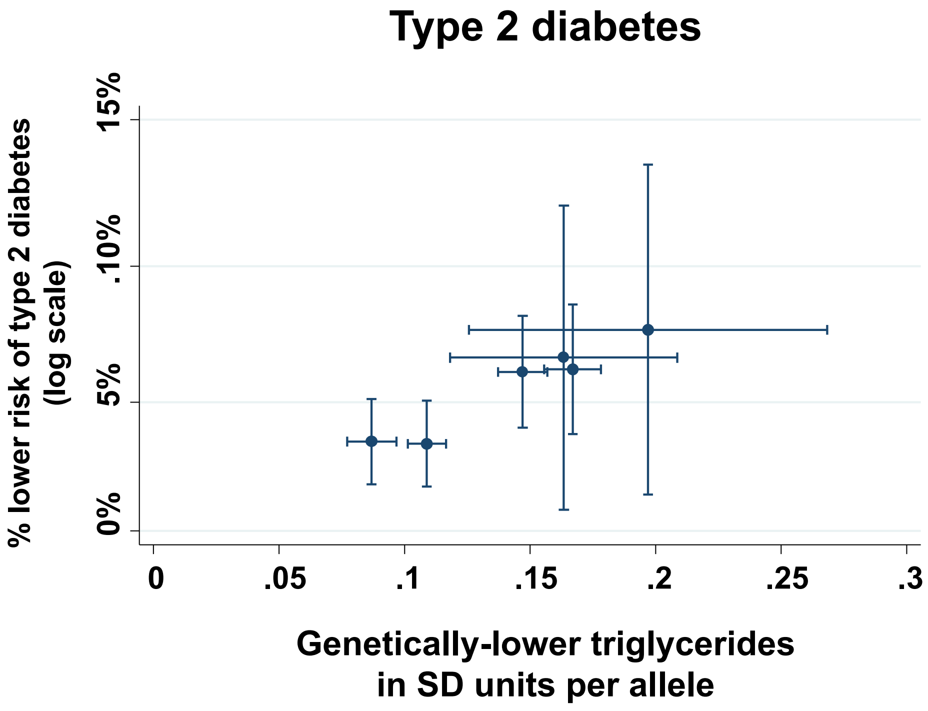

© 2018 Lotta LA et al. *JAMA Cardiol.*

eFigure 4. Associations with lipid traits in 2 × 2 factorial genetic analyses

The figure shows associations with lipid traits expressed in standardized units for each group compared to the reference group. Data on lipid traits were from the EPIC-Norfolk study and the EPIC-InterAct study subcohort. Median values and interquartile ranges for lipid levels are from the EPIC-Norfolk study. Abbreviations: N, number of participants; CI, confidence interval; LDL-C, low-density lipoprotein cholesterol; LPL, lipoprotein lipase; SD, standard deviation; IQR, interquartile range.

Genotype category	Proxy for	Outcome	LDL-C median (IQR) mmol/L	Triglycerides median (IQR) mmol/L	Beta (95% CI)	N	p-value
Reference:<br>LDL-C lowering score ≤ median<br>Triglyceride-lowering *LPL* score ≤ median	Placebo	LDL-C<br>Triglycerides	4.1<br>(3.5 – 4.8)	1.7<br>(1.2 – 2.4)	Reference<br>Reference	8,098<br>8,098	Reference<br>Reference
Genetically-lower triglycerides via *LPL* only:<br>LDL-C lowering score ≤ median<br>Triglyceride-lowering *LPL* score > median	LPL-enhancing Therapy only	LDL-C<br>Triglycerides	4.1<br>(3.5 – 4.8)	1.5<br>(1.1 – 2.2)	-0.02 (-0.05, 0.01)<br>-0.17 (-0.20, -0.14)	7,816<br>7,816	0.27<br>2.3×10^{-27}
Genetically-lower LDL-C only:<br>LDL-C lowering score > median<br>Triglyceride-lowering *LPL* score ≤ median	LDL-C lowering Therapy only	LDL-C<br>Triglycerides	3.7<br>(3.1 – 4.3)	1.6<br>(1.1 – 2.2)	-0.46 (-0.49, -0.43)<br>-0.12 (-0.15, -0.09)	8,239<br>8,239	2.7×10^{-200}<br>6.1×10^{-15}
Both exposures:<br>LDL-C lowering score > median<br>Triglyceride-lowering *LPL* score > median	LPL-enhancing and LDL-C lowering therapy	LDL-C<br>Triglycerides	3.7<br>(3.1 – 4.3)	1.5<br>(1.0 – 2.1)	-0.47 (-0.50, -0.44)<br>-0.25 (-0.28, -0.22)	7,674<br>7,674	1.7×10^{-204}<br>1.1×10^{-58}

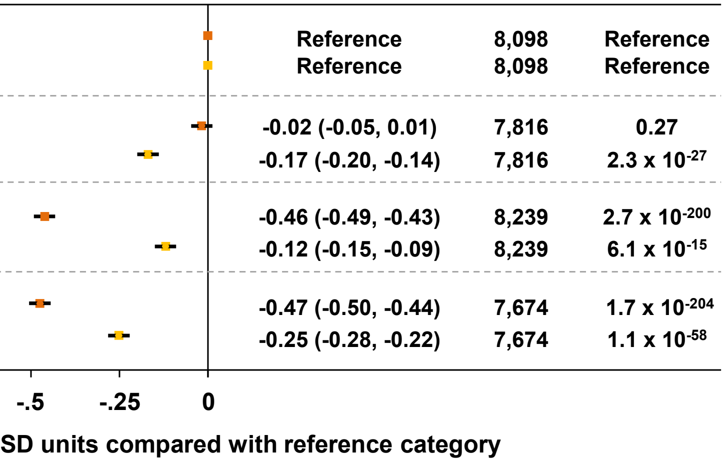

© 2018 Lotta LA et al. *JAMA Cardiol.*

eFigure 5. Associations of triglyceride-lowering alleles in *LPL* with risk of coronary artery disease and type 2 diabetes in individuals above or below the median of the population distribution of genetic variants at *NPC1L1* or *PCSK9*

Analyses include individual-level genetic data from 390,470 participants of the UK Biobank, EPIC-Norfolk and EPIC-InterAct studies. Abbreviations: LPL, lipoprotein lipase; LDL-C, low-density lipoprotein cholesterol; *NPC1L1*, Niemann-Pick C1-Like 1; *PCSK9*, Proprotein convertase subtilisin/kexin type 9; SD, standard deviation; OR, odds ratio.

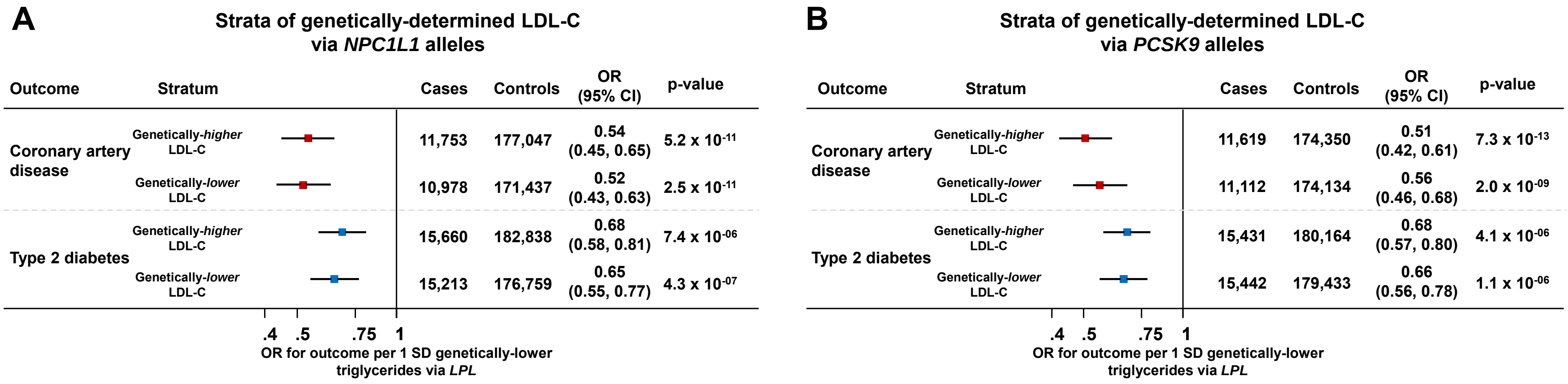

© 2018 Lotta LA et al. *JAMA Cardiol.*

eFigure 6. Lipid levels and cardiometabolic outcomes risk in quintiles of the population distribution of genetic variants at 58 LDL-C–associated genetic loci

The figure shows associations with lipid traits (left) and cardio-metabolic outcomes risk (right) for individuals in a given quintile compared to the bottom quintile (Q1). Data are from the UK Biobank, EPIC-Norfolk and EPIC-InterAct studies. Median values and interquartile ranges for lipid levels are from the EPIC-Norfolk study. Abbreviations: N, number of participants; CI, confidence interval; LDL-C, low-density lipoprotein cholesterol; SD, standard deviation; OR, odds ratio; Q, quintile.

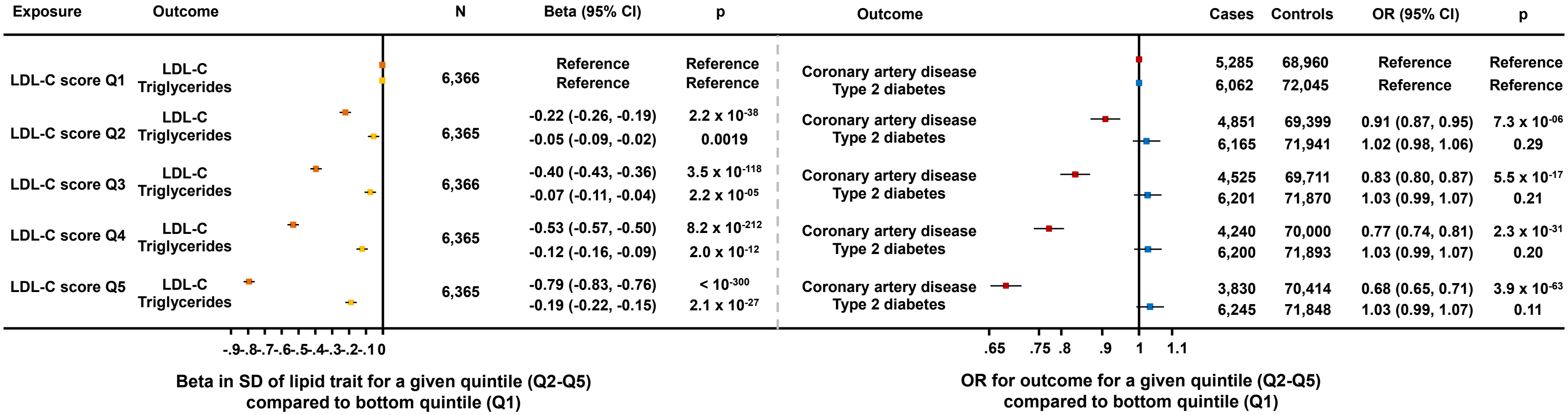

Exposure	Outcome	N	Beta (95% CI)	p
LDL-C score Q1	LDL-C	6,366	Reference	Reference
	Triglycerides		Reference	Reference
LDL-C score Q2	LDL-C	6,365	-0.22 (-0.26, -0.19)	2.2×10^{-38}
	Triglycerides		-0.05 (-0.09, -0.02)	0.0019
LDL-C score Q3	LDL-C	6,366	-0.40 (-0.43, -0.36)	3.5×10^{-118}
	Triglycerides		-0.07 (-0.11, -0.04)	2.2×10^{-05}
LDL-C score Q4	LDL-C	6,365	-0.53 (-0.57, -0.50)	8.2×10^{-212}
	Triglycerides		-0.12 (-0.16, -0.09)	2.0×10^{-12}
LDL-C score Q5	LDL-C	6,365	-0.79 (-0.83, -0.76)	$< 10^{-300}$
	Triglycerides		-0.19 (-0.22, -0.15)	2.1×10^{-27}

Outcome	Cases	Controls	OR (95% CI)	p
Coronary artery disease	5,285	68,960	Reference	Reference
Type 2 diabetes	6,062	72,045	Reference	Reference
Coronary artery disease	4,851	69,399	0.91 (0.87, 0.95)	7.3×10^{-06}
Type 2 diabetes	6,165	71,941	1.02 (0.98, 1.06)	0.29
Coronary artery disease	4,525	69,711	0.83 (0.80, 0.87)	5.5×10^{-17}
Type 2 diabetes	6,201	71,870	1.03 (0.99, 1.07)	0.21
Coronary artery disease	4,240	70,000	0.77 (0.74, 0.81)	2.3×10^{-31}
Type 2 diabetes	6,200	71,893	1.03 (0.99, 1.07)	0.20
Coronary artery disease	3,830	70,414	0.68 (0.65, 0.71)	3.9×10^{-63}
Type 2 diabetes	6,245	71,848	1.03 (0.99, 1.07)	0.11

© 2018 Lotta LA et al. *JAMA Cardiol.*

eFigure 7. Association with risk of coronary artery disease of LDL-C–lowering genetic variants at *ANGPTL3* and other loci

The figure shows associations of LDL-C lowering variants at *NPC1L1*, *HMGCR*, *PCSK9*, 58 LDL-C associated genomic regions or *ANGPTL3*. Estimates for *NPC1L1*, *HMGCR*, *PCSK9*, 58 LDL-C associated genomic regions are from UK Biobank and CARDIoGRAMplusC4D, while estimates for *ANGPTL3* are from a meta-analysis of published genetic association studies (see eNote 4, eTable 8 and eFigure 8). The top graph shows a comparison of the estimates for *NPC1L1*, *HMGCR*, *PCSK9* variants and the 58-variant LDL-C genetic score. The bottom panel shows a comparison of the estimates for *ANGPTL3* variants and the 58-variant LDL-C genetic score.

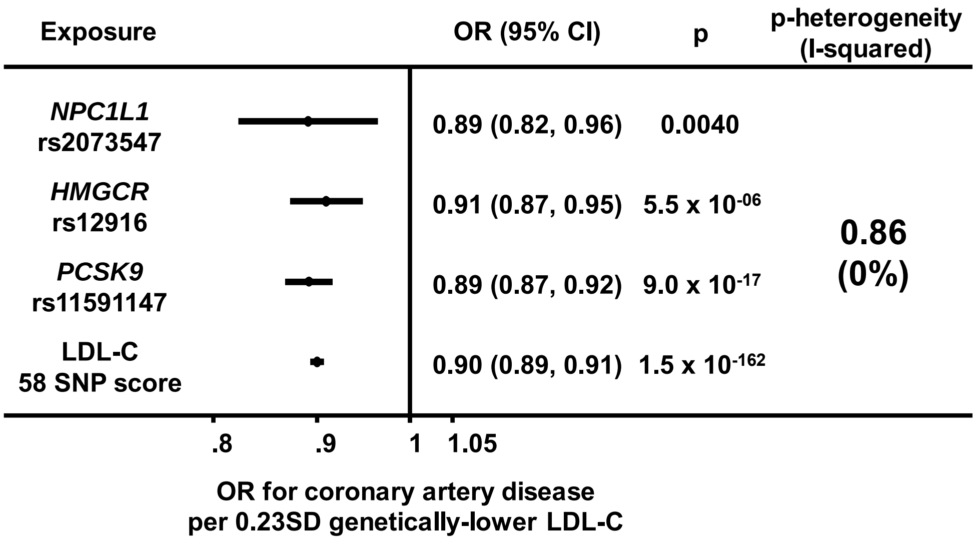

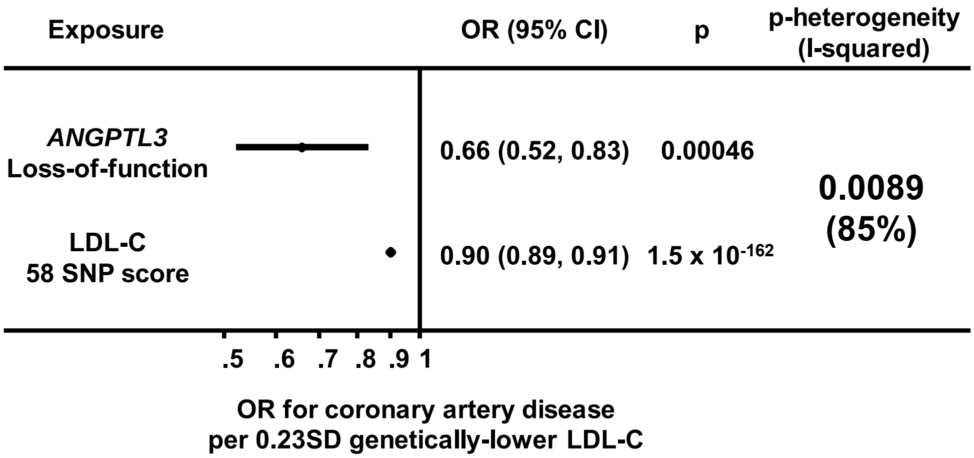

© 2018 Lotta LA et al. *JAMA Cardiol.*

eFigure 8. Meta-analysis of genetic association studies of *ANGPTL3* rare loss-of-function variants and risk of coronary artery disease

Data are from previously published studies including 58,399 coronary artery disease cases and 305,796 controls.[23,24]

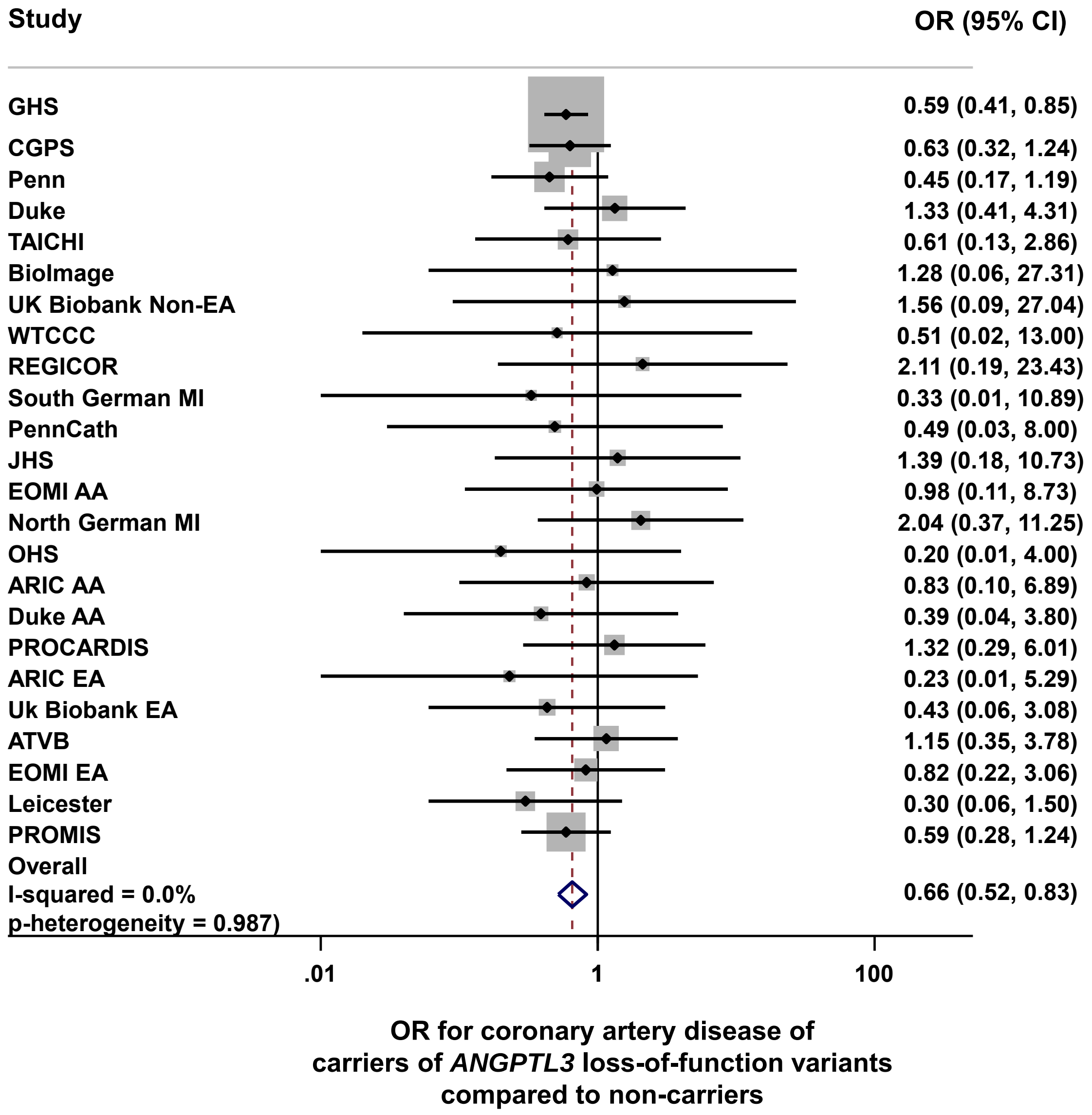

© 2018 Lotta LA et al. *JAMA Cardiol.*

eTable 1. Data sources and participating studies

Summary of the studies participating in the different analyses of the manuscript.

Analysis	Outcome	Total cases, N	Total controls (for disease outcomes) or participants (for continuous traits), N	Participating study	Study cases, N	Study non-cases (for case-control studies) or participants (for continuous traits studies), N	PubMed ID for cohort description	Website (URL)
Non-stratified analyses of summary-level genetic data (**eFigure 1A**)	Body mass index	-	672,505	UK Biobank	-	350,803	25826379	http://www.ukbiobank.ac.uk/
				GIANT Consortium	-	321,702	25673413	https://www.broadinstitute.org/collaboration/giant/index.php/GIANT_consortium
	Waist-to-hip ratio adjusted for body mass index	-	559,817	UK Biobank	-	350,051	25826379	http://www.ukbiobank.ac.uk/
				GIANT Consortium	-	209,766	25673412	https://www.broadinstitute.org/collaboration/giant/index.php/GIANT_consortium
	Low-density lipoprotein cholesterol	-	188,577	Global Lipids Genetics Consortium	-	188,577	24097068	http://csg.sph.umich.edu//abecasis/public/lipids2013/
	Triglycerides	-	188,577	Global Lipids Genetics Consortium	-	188,577	24097068	http://csg.sph.umich.edu//abecasis/public/lipids2013/
	Fasting plasma glucose	-	133,010	MAGIC Consortium	-	133,010	22885924, 22581228	http://www.magicinvestigators.org/
	Fasting insulin	-	108,557	MAGIC Consortium	-	108,557	22885924, 22581228	http://www.magicinvestigators.org/
	Systolic blood pressure	-	351,354	UK Biobank	-	351,354	25826379	http://www.ukbiobank.ac.uk/
	Diastolic blood pressure	-	351,361	UK Biobank	-	351,361	25826379	http://www.ukbiobank.ac.uk/
	Type 2 diabetes	63,859	457,289	EPIC-InterAct	9,400	11,593	21717116	http://www.inter-act.eu/
				UK Biobank	19,619	330,715	25826379	http://www.ukbiobank.ac.uk/
				DIAGRAM	34,840	114,981	22885922	http://diagram-consortium.org/
	Coronary artery disease	79,304	457,071	UK Biobank	18,503	333,567	25826379	http://www.ukbiobank.ac.uk/
				CARDIoGRAMplusC4D Consortium	60,801	123,504	26343387	http://www.cardiogramplusc4d.org/
Factorial or	Low-density	-	31,827	EPIC-Norfolk	-	19,157	10466767	http://www.srl.cam.ac.uk/epic/

© 2018 Lotta LA et al. *JAMA Cardiol.*

stratified genetic analyses of individual-level genetic data (**eFigure 1B-C**)	lipoprotein cholesterol			EPIC-InterAct subcohort	-	12,670	21717116	http://www.inter-act.eu/
	Triglycerides	-	31,827	EPIC-Norfolk	-	19,157	10466767	http://www.srl.cam.ac.uk/epic/
				EPIC-InterAct subcohort	-	12,670	21717116	http://www.inter-act.eu/
	Type 2 diabetes	30,873	359,597	UK Biobank	19,619	330,715	25826379	http://www.ukbiobank.ac.uk/
				EPIC-InterAct	9,400	11,593	21717116	http://www.inter-act.eu/
				EPIC-Norfolk	1,854	17,289	10466767	http://www.srl.cam.ac.uk/epic/
	Coronary artery disease	22,731	348,484	UK Biobank	18,503	333,567	25826379	http://www.ukbiobank.ac.uk/
				EPIC-Norfolk	4,228	14,917	10466767	http://www.srl.cam.ac.uk/epic/

Abbreviations: N, number of participants.

© 2018 Lotta LA et al. *JAMA Cardiol.*

eTable 2. List of genetic variants in *LPL* and LDL cholesterol pathways investigated in this study

Genetic score	dbSNP ID	Chromosome	Position	Effect allele[a]	Other allele	Phenotype	Beta	SE	Reference[b]
Lower triglycerides via *LPL*	rs268	8	19813529	A	G	ln-triglycerides	-0.1971	0.0364	This study[c]
	rs328	8	19819724	G	C	ln-triglycerides	-0.167	0.0058	24097068
	rs1801177	8	19805708	G	A	ln-triglycerides	-0.1635	0.0231	24097068
	rs10096633	8	19830921	T	C	ln-triglycerides	-0.1471	0.005	24097068
	rs301	8	19816934	C	T	ln-triglycerides	-0.1089	0.0039	24097068
	rs326	8	19819439	G	A	ln-triglycerides	-0.0869	0.005	24097068
Lower LDL-C via 58 genetic regions	rs9987289	8	9183358	A	G	LDL-C	-0.0714	0.0066	24097068
	rs3764261	16	56993324	A	C	LDL-C	-0.0528	0.0042	24097068
	rs2479409	1	55504650	A	G	LDL-C	-0.0642	0.0041	24097068
	rs629301	1	109818306	G	T	LDL-C	-0.1669	0.0049	24097068
	rs1367117	2	21263900	G	A	LDL-C	-0.1186	0.004	24097068
	rs4299376	2	44072576	T	G	LDL-C	-0.0812	0.0045	24097068
	rs3757354	6	16127407	T	C	LDL-C	-0.0382	0.0044	24097068
	rs1800562	6	26093141	A	G	LDL-C	-0.0615	0.008	24097068
	rs1564348	6	160578860	T	C	LDL-C	-0.0481	0.005	24097068
	rs11136341	8	145043543	A	G	LDL-C	-0.0447	0.0062	24097068
	rs635634	9	136155000	C	T	LDL-C	-0.0772	0.0055	24097068
	rs11220462	11	126243952	G	A	LDL-C	-0.059	0.0059	24097068
	rs8017377	14	24883887	G	A	LDL-C	-0.0303	0.0038	24097068
	rs7206971	17	45425115	G	A	LDL-C	-0.0292	0.0055	24097068
	rs6511720	19	11202306	T	G	LDL-C	-0.2209	0.0061	24097068
	rs4420638	19	45422946	A	G	LDL-C	-0.2251	0.0077	24097068
	rs6029526	20	39672618	T	A	LDL-C	-0.0436	0.0052	24097068
	rs12027135	1	25775733	A	T	LDL-C	-0.03	0.0038	24097068
	rs2642442	1	220973563	C	T	LDL-C	-0.036	0.0054	24097068
	rs514230	1	234858597	A	T	LDL-C	-0.0364	0.0054	24097068
	rs12916	5	74656539	T	C	LDL-C	-0.0733	0.0038	24097068
	rs6882076	5	156390297	T	C	LDL-C	-0.0456	0.0038	24097068
	rs3177928	6	32412435	G	A	LDL-C	-0.0452	0.0052	24097068

© 2018 Lotta LA et al. *JAMA Cardiol.*

	rs9488822	6	116312893	T	A	LDL-C	-0.0311	0.0054	24097068
	rs12670798	7	21607352	T	C	LDL-C	-0.0344	0.0043	24097068
	rs2072183	7	44579180	G	C	LDL-C	-0.0386	0.0047	24097068
	rs2081687	8	59388565	C	T	LDL-C	-0.0311	0.0054	24097068
	rs2255141	10	113933886	G	A	LDL-C	-0.0299	0.004	24097068
Lower LDL-C via 58 genetic regions	rs11065987	12	112072424	G	A	LDL-C	-0.0269	0.0038	24097068
	rs1169288	12	121416650	A	C	LDL-C	-0.0375	0.004	24097068
	rs2000999	16	72108093	G	A	LDL-C	-0.065	0.0046	24097068
	rs10401969	19	19407718	C	T	LDL-C	-0.1184	0.0072	24097068
	rs2902940	20	39091487	G	A	LDL-C	-0.0274	0.0041	24097068
	rs2131925	1	63025942	G	T	LDL-C	-0.0489	0.0039	24097068
	rs2954029	8	126490972	T	A	LDL-C	-0.0564	0.0036	24097068
	rs174546	11	61569830	T	C	LDL-C	-0.0512	0.0038	24097068
	rs964184	11	116648917	C	G	LDL-C	-0.0855	0.0078	24097068
	rs12748152	1	27138393	C	T	LDL-C	-0.0499	0.0066	24097068
	rs267733	1	150958836	G	A	LDL-C	-0.0331	0.0053	24097068
	rs2710642	2	63149557	G	A	LDL-C	-0.0239	0.0038	24097068
	rs10490626	2	118835841	A	G	LDL-C	-0.0508	0.0069	24097068
	rs2030746	2	121309488	C	T	LDL-C	-0.0214	0.0038	24097068
	rs1250229	2	216304384	T	C	LDL-C	-0.0243	0.0042	24097068
	rs7640978	3	32533010	T	C	LDL-C	-0.0392	0.0069	24097068
	rs17404153	3	132163200	T	G	LDL-C	-0.0336	0.0054	24097068
	rs4530754	5	122855416	G	A	LDL-C	-0.0275	0.0036	24097068
	rs4722551	7	25991826	T	C	LDL-C	-0.0391	0.0049	24097068
	rs10102164	8	55421614	G	A	LDL-C	-0.0316	0.0045	24097068
	rs4942486	13	32953388	C	T	LDL-C	-0.0243	0.0037	24097068
	rs1801689	17	64210580	A	C	LDL-C	-0.1028	0.0139	24097068
	rs364585	20	12962718	A	G	LDL-C	-0.0249	0.0038	24097068
	rs2328223	20	17845921	A	C	LDL-C	-0.0299	0.005	24097068
	rs5763662	22	30378703	C	T	LDL-C	-0.0767	0.0121	24097068
	rs11563251	2	234679384	C	T	LDL-C	-0.0345	0.0062	24097068

© 2018 Lotta LA et al. *JAMA Cardiol.*

	rs3780181	9	2640759	G	A	LDL-C	-0.0445	0.0074	24097068
	rs314253	17	7091650	C	T	LDL-C	-0.0242	0.0038	24097068
	rs4253772	22	46627603	C	T	LDL-C	-0.0313	0.006	24097068
	rs6831256	4	3473139	A	G	LDL-C	-0.0188	0.0038	24097068
Lower LDL-C via *NPC1L1*	rs217386	7	44600695	A	G	LDL-C	-0.0363	0.0038	24097068
	rs2073547	7	44582331	A	G	LDL-C	-0.0485	0.0049	24097068
	rs7791240	7	44602589	T	C	LDL-C	-0.0425	0.0065	24097068
	rs10234070	7	44537696	C	T	LDL-C	-0.0295	0.0059	24097068
	rs2300414	7	44682938	G	A	LDL-C	-0.0353	0.008	24097068
Lower LDL-C via *HMGCR*	rs12916	5	74656539	T	C	LDL-C	-0.0733	0.0038	24097068
	rs17238484	5	74648496	G	T	LDL-C	-0.0627	0.0062	24097068
Lower LDL-C via *HMGCR*	rs5909	5	74656175	G	A	LDL-C	-0.0617	0.0088	24097068
	rs2303152	5	74641707	G	A	LDL-C	-0.0423	0.0064	24097068
	rs10066707	5	74560579	G	A	LDL-C	-0.0497	0.0054	24097068
	rs2006760	5	74562029	C	G	LDL-C	-0.0533	0.0076	24097068
Lower LDL-C via *PCSK9*	rs11206510	1	55496039	C	T	LDL-C	-0.0831	0.005	24097068
	rs2479409	1	55504650	A	G	LDL-C	-0.0642	0.0041	24097068
	rs2149041	1	55502137	C	G	LDL-C	-0.0636	0.0049	24097068
	rs2479394	1	55486064	A	G	LDL-C	-0.0386	0.0041	24097068
	rs10888897	1	55513061	T	C	LDL-C	-0.0507	0.0042	24097068
	rs7552841	1	55518752	C	T	LDL-C	-0.0368	0.0044	24097068
	rs562556	1	55524237	G	A	LDL-C	-0.064	0.0066	24097068

Abbreviations: SE, standard error; *LPL*, lipoprotein lipase; LDL-C, low-density lipoprotein cholesterol; *HMGCR*, 3-Hydroxy-3-Methylglutaryl-CoA Reductase; *NPC1L1*, Niemann-Pick C1-Like 1; *PCSK9*, Proprotein convertase subtilisin/kexin type 9.

a The effect allele is the lipid-lowering allele.

b PubMed ID of the original manuscript from which beta coefficients and standard errors are derived.

c Estimated in EPIC-Norfolk.

© 2018 Lotta LA et al. *JAMA Cardiol.*

eTable 3. Linkage disequilibrium between *LPL* genetic variants included in the analysis

rsID	rs268	rs328	rs1801177	rs10096633	rs301	rs326
**rs268**	1	0.002	0	0.003	0.002	0.001
**rs328**	0.002	1	0.002	0.736	0.171	0.308
**rs1801177**	0	0.002	1	0.002	0.009	0.005
**rs10096633**	0.003	0.736	0.002	1	0.098	0.418
**rs301**	0.002	0.171	0.009	0.098	1	0.243
**rs326**	0.001	0.308	0.005	0.418	0.243	1

All measures of LD are in R^2 and were derived from the LDlink software using five European ancestry populations from phase 3 of the 1000 genomes project

© 2018 Lotta LA et al. *JAMA Cardiol.*

eTable 4. Sensitivity analysis of the association between triglyceride-lowering *LPL* alleles and risk of coronary artery disease and type 2 diabetes using only 3 variants with very low reciprocal linkage disequilibrium

Estimates were nearly identical to those obtained with all six genetic variants (eFigure 2), but less precise (R^2<0.01; rs268, rs328, rs1801177).

Outcome	Cases	Controls	OR (95% CI)	p-value
Coronary artery disease	79,304	457,071	0.61 (0.54, 0.69)	4.73×10^{-16}
Type 2 diabetes	63,859	457,289	0.68 (0.59, 0.77)	4.53×10^{-09}

Odds ratios are per SD genetically-lower triglycerides via *LPL*.
Abbreviations: CAD, coronary artery disease; T2D, type 2 diabetes; SD, standard deviation; SE, standard error; OR, odds ratio; CI, confidence interval

© 2018 Lotta LA et al. *JAMA Cardiol.*

eTable 5. Triglyceride-lowering alleles in *LPL* and risk of coronary artery disease and type 2 diabetes

dbSNP rsID	Genomic coordinate, chromosome and position	Effect allele[a] / other allele	Effect allele frequency, mean (range)[b]	Beta (SE) per allele in standardized triglyceride levels[c]	OR of coronary artery disease (95% CI) per allele[d]	p-value	OR of type 2 diabetes (95% CI) per allele[e]	p-value
rs268	chr8: 19813529	A / G	0.98 (0.98, 0.98)	-0.197 (0.036)	0.88 (0.83, 0.93)	6.4×10^{-06}	0.92 (0.86, 0.99)	0.017
rs328	chr8: 19819724	G / C	0.11 (0.11, 0.12)	-0.167 (0.006)	0.94 (0.91, 0.96)	3.9×10^{-08}	0.94 (0.91, 0.96)	1.0×10^{-06}
rs1801177	chr8: 19805708	G / A	0.98 (0.98, 0.98)	-0.164 (0.023)	0.88 (0.83, 0.93)	2.9×10^{-06}	0.93 (0.88, 0.99)	0.026
rs10096633	chr8: 19830921	T / C	0.13 (0.12, 0.14)	-0.147 (0.005)	0.94 (0.92, 0.96)	1.7×10^{-08}	0.94 (0.92, 0.96)	2.7×10^{-08}
rs301	chr8: 19816934	C / T	0.24 (0.24, 0.25)	-0.109 (0.004)	0.95 (0.92, 0.97)	5.6×10^{-05}	0.97 (0.95, 0.98)	7.6×10^{-05}
rs326	chr8: 19819439	G / A	0.30 (0.29, 0.31)	-0.087 (0.005)	0.95 (0.93, 0.96)	1.2×10^{-11}	0.97 (0.95, 0.98)	4.8×10^{-05}

Abbreviations: SE, standard error; OR, odds ratio; CI, confidence interval.
a The effect allele is the triglyceride-lowering allele.
b In the EPIC-Norfolk, EPIC-InterAct and UK Biobank studies.
c Data from the Global Lipids Genetics Consortium, except rs268 for which data is from EPIC-Norfolk.
d Data from the CARDIoGRAMplusC4D consortium and the UK Biobank study, except rs301 for which data is from UK Biobank.
e Data from EPIC-InterAct, DIAGRAM and UK Biobank, except rs268 for which data is from EPIC-InterAct and UK Biobank.

© 2018 Lotta LA et al. *JAMA Cardiol.*

eTable 6. Association with type 2 diabetes of triglyceride-lowering genetic variants at the *LPL* gene or at several triglyceride-associated regions studied by White et al[35]

Exposure	Reference[a]	OR for type 2 diabetes (95% CI)	Heterogeneity in effect estimates[c], p-value
Triglyceride-lowering alleles in *LPL*	This study	0.69 (0.62, 0.76)	Reference
140 triglyceride-lowering alleles at multiple genetic loci in inverse variance weighted Mendelian randomisation analyses[b]	27487401	0.99 (0.90, 1.09)	1.6×10^{-07}
140 triglyceride-lowering alleles at multiple genetic loci in multivariable Mendelian randomisation analyses[b]	27487401	1.00 (0.86, 1.16)	4.5×10^{-05}
140 triglyceride-lowering alleles at multiple genetic loci in Egger Mendelian randomisation analyses[b]	27487401	1.20 (1.05, 1.39)	2.3×10^{-10}

Abbreviations: OR, odds ratio; CI, confidence interval; LPL, lipoprotein lipase.

a PubMed manuscript ID.

b Inverse variance weighted Mendelian randomisation is a primary analysis method in Mendelian randomisation analyses; multi-variable Mendelian randomisation is an analysis method that adjusts for estimates on other traits (i.e. HDL and LDL cholesterol in this case); Egger Mendelian randomisation is a sensitivity analysis method that is robust to directional pleiotropy.

c Comparison between the estimate for *LPL* alleles from this study (reference group) and each of the two estimates from White and colleagues using 140 triglyceride-lowering alleles from multiple genetic loci.

eTable 7. Sensitivity analysis of the association between triglyceride-lowering *LPL* alleles and risk of coronary artery disease and type 2 diabetes in people above or below the median of the population distribution of 22 LDL-C–lowering variants associated with LDL-C but not triglyceride levels

Estimates were nearly identical to those obtained with all 58 LDL-C genetic variants (Figure 2A).

Exposure	Outcome	Subgroup of 22-variant LDL-C lowering genetic score	Cases / Controls	OR (95% CI)	p-value
Triglyceride-lowering *LPL*-alleles	Coronary artery disease	Below median	12,079 / 173,530	0.60 (0.50, 0.71)	2.3×10^{-08}
		Above median	10,652 / 174,954	0.47 (0.39, 0.57)	1.7×10^{-14}
	Type 2 diabetes	Below median	15,366 / 179,897	0.72 (0.61, 0.85)	9.5×10^{-05}
		Above median	15,507 / 179,700	0.62 (0.53, 0.74)	3.6×10^{-08}

Abbreviations: OR, odds ratio; CI, confidence interval; LDL-C, low-density lipoprotein cholesterol.

© 2018 Lotta LA et al. *JAMA Cardiol.*

eTable 8. Heterogeneity in estimates of the association with coronary disease of *ANGPTL3* loss-of-function variants and LDL-C–lowering polygenic score in sensitivity analyses

***ANGPTL3* analysis**	***ANGPTL3* estimate, OR (95% CI)[a]**	**LDL-C lowering score analysis**	**LDL-C lowering score estimate, OR (95% CI)[a]**	**Heterogeneity p-value[b]**
Main	0.66 (0.52-0.83)	22-variant score[d]	0.89 (0.88-0.90)	0.011
Main	0.66 (0.52-0.83)	White IVW[e]	0.91 (0.89-0.92)	0.0076
Main	0.66 (0.52-0.83)	White multivariable[f]	0.91 (0.89-0.93)	0.0068
Main	0.66 (0.52-0.83)	White Egger-MR[g]	0.89 (0.86-0.91)	0.013
PennCath[c] excluded	0.66 (0.52-0.83)	58-variant score[h]	0.90 (0.89-0.91)	0.0096
PennCath[c] excluded	0.66 (0.52-0.83)	22-variant score[d]	0.89 (0.88-0.90)	0.011
PennCath[c] excluded	0.66 (0.52-0.83)	White IVW[e]	0.91 (0.89-0.92)	0.0082
PennCath[c] excluded	0.66 (0.52-0.83)	White multivariable[f]	0.91 (0.89-0.93)	0.0074
PennCath[c] excluded	0.66 (0.52-0.83)	White Egger-MR[g]	0.89 (0.86-0.91)	0.014

Abbreviations: OR, odds ratio; CI, confidence interval; LDL-C, low-density lipoprotein cholesterol; IVW, inverse variance weighted method; MR, Mendelian randomization.

a Odds ratio for coronary artery disease per 0.23 SD genetically-lower LDL-C.

b Heterogeneity p-value for comparison of effect estimates between *ANGPTL3* variants and LDL-C lowering score analysis.

c Sensitivity analysis excluding estimates from PennCath study[24] to account for any possible overlap with the Penn Medicine Biobank.[23]

d Estimate from UK Biobank and CARDIoGRAMplusC4D of the association of 22 variants associated with LDL-C ($p<5\times10^{-08}$) but not triglyceride ($p>0.05$) levels in GLGC.[12]

e Estimate from White and colleagues of the association with coronary disease of 130 single-nucleotide polymorphisms associated with LDL-C – inverse variance weighted method.[35]

f Estimate from White and colleagues of the association with coronary disease of 130 single-nucleotide polymorphisms associated with LDL-C – multivariable Mendelian randomization method (adjusted for HDL-C and triglycerides).[35]

g Estimate from White and colleagues of the association with coronary disease of 130 single-nucleotide polymorphisms associated with LDL-C – Egger Mendelian randomization method.[35]

© 2018 Lotta LA et al. *JAMA Cardiol.*

eReferences.

1. InterAct Consortium, Langenberg C, Sharp S, et al. Design and cohort description of the InterAct Project: an examination of the interaction of genetic and lifestyle factors on the incidence of type 2 diabetes in the EPIC Study. *Diabetologia.* Sep 2011;54(9):2272-2282.

2. Riboli E, Kaaks R. The EPIC Project: rationale and study design. European Prospective Investigation into Cancer and Nutrition. *International journal of epidemiology.* 1997;26 Suppl 1:S6-14.

3. Sudlow C, Gallacher J, Allen N, et al. UK biobank: an open access resource for identifying the causes of a wide range of complex diseases of middle and old age. *PLoS medicine.* Mar 2015;12(3):e1001779.

4. Eastwood SV, Mathur R, Atkinson M, et al. Algorithms for the Capture and Adjudication of Prevalent and Incident Diabetes in UK Biobank. *PloS one.* 2016;11(9):e0162388.

5. Klarin D, Zhu QM, Emdin CA, et al. Genetic analysis in UK Biobank links insulin resistance and transendothelial migration pathways to coronary artery disease. *Nature genetics.* Sep 2017;49(9):1392-1397.

6. Nelson CP, Goel A, Butterworth AS, et al. Association analyses based on false discovery rate implicate new loci for coronary artery disease. *Nature genetics.* Jul 17 2017.

7. Bycroft C, Freeman C, Petkova D, et al. Genome-wide genetic data on ~500,000 UK Biobank participants. *bioRxiv.* 2017. Doi: https://doi.org/10.1101/166298.

8. Day N, Oakes S, Luben R, et al. EPIC-Norfolk: study design and characteristics of the cohort. European Prospective Investigation of Cancer. *British journal of cancer.* Jul 1999;80 Suppl 1:95-103.

9. Lotta LA, Gulati P, Day FR, et al. Integrative genomic analysis implicates limited peripheral adipose storage capacity in the pathogenesis of human insulin resistance. *Nature genetics.* Jan 2017;49(1):17-26.

10. Morris AP, Voight BF, Teslovich TM, et al. Large-scale association analysis provides insights into the genetic architecture and pathophysiology of type 2 diabetes. *Nature genetics.* Sep 2012;44(9):981-990.

11. Nikpay M, Goel A, Won HH, et al. A comprehensive 1,000 Genomes-based genome-wide association meta-analysis of coronary artery disease. *Nature genetics.* Oct 2015;47(10):1121-1130.

12. Global Lipids Genetics Consortium, Willer CJ, Schmidt EM, et al. Discovery and refinement of loci associated with lipid levels. *Nature genetics.* Nov 2013;45(11):1274-1283.

13. Locke AE, Kahali B, Berndt SI, et al. Genetic studies of body mass index yield new insights for obesity biology. *Nature.* Feb 12 2015;518(7538):197-206.

© 2018 Lotta LA et al. *JAMA Cardiol.*

14. Shungin D, Winkler TW, Croteau-Chonka DC, et al. New genetic loci link adipose and insulin biology to body fat distribution. *Nature.* Feb 12 2015;518(7538):187-196.

15. Scott RA, Lagou V, Welch RP, et al. Large-scale association analyses identify new loci influencing glycemic traits and provide insight into the underlying biological pathways. *Nature genetics.* Sep 2012;44(9):991-1005.

16. Manning AK, Hivert MF, Scott RA, et al. A genome-wide approach accounting for body mass index identifies genetic variants influencing fasting glycemic traits and insulin resistance. *Nature genetics.* Jun 2012;44(6):659-669.

17. Khera AV, Won HH, Peloso GM, et al. Association of Rare and Common Variation in the Lipoprotein Lipase Gene With Coronary Artery Disease. *JAMA : the journal of the American Medical Association.* Mar 07 2017;317(9):937-946.

18. Davey Smith G, Ebrahim S. What can mendelian randomisation tell us about modifiable behavioural and environmental exposures? *Bmj.* May 7 2005;330(7499):1076-1079.

19. Ference BA, Majeed F, Penumetcha R, Flack JM, Brook RD. Effect of naturally random allocation to lower low-density lipoprotein cholesterol on the risk of coronary heart disease mediated by polymorphisms in NPC1L1, HMGCR, or both: a 2 x 2 factorial Mendelian randomization study. *Journal of the American College of Cardiology.* Apr 21 2015;65(15):1552-1561.

20. Ference BA, Robinson JG, Brook RD, et al. Variation in PCSK9 and HMGCR and Risk of Cardiovascular Disease and Diabetes. *The New England journal of medicine.* Dec 01 2016;375(22):2144-2153.

21. Burgess S, Dudbridge F, Thompson SG. Combining information on multiple instrumental variables in Mendelian randomization: comparison of allele score and summarized data methods. *Statistics in medicine.* May 20 2016;35(11):1880-1906.

22. Musunuru K, Pirruccello JP, Do R, et al. Exome sequencing, ANGPTL3 mutations, and familial combined hypolipidemia. *The New England journal of medicine.* Dec 02 2010;363(23):2220-2227.

23. Dewey FE, Gusarova V, Dunbar RL, et al. Genetic and Pharmacologic Inactivation of ANGPTL3 and Cardiovascular Disease. *The New England journal of medicine.* Jul 20 2017;377(3):211-221.

24. Stitziel NO, Khera AV, Wang X, et al. ANGPTL3 Deficiency and Protection Against Coronary Artery Disease. *Journal of the American College of Cardiology.* Apr 25 2017;69(16):2054-2063.

25. Silverman MG, Ference BA, Im K, et al. Association Between Lowering LDL-C and Cardiovascular Risk Reduction Among Different Therapeutic Interventions: A Systematic Review and Meta-analysis. *JAMA : the journal of the American Medical Association.* Sep 27 2016;316(12):1289-1297.

© 2018 Lotta LA et al. *JAMA Cardiol.*

26. Jarcho JA, Keaney JF, Jr. Proof That Lower Is Better--LDL Cholesterol and IMPROVE-IT. *The New England journal of medicine.* Jun 18 2015;372(25):2448-2450.

27. Gaudet D, Brisson D, Tremblay K, et al. Targeting APOC3 in the familial chylomicronemia syndrome. *The New England journal of medicine.* Dec 4 2014;371(23):2200-2206.

28. Gaudet D, Alexander VJ, Baker BF, et al. Antisense Inhibition of Apolipoprotein C-III in Patients with Hypertriglyceridemia. *The New England journal of medicine.* Jul 30 2015;373(5):438-447.

29. TG and HDL Working Group of the Exome Sequencing Project, National Heart, Lung, and Blood Institute, et al. Loss-of-function mutations in APOC3, triglycerides, and coronary disease. The New England journal of medicine. Jul 3 2014;371(1):22-31.

30. Jorgensen AB, Frikke-Schmidt R, Nordestgaard BG, Tybjaerg-Hansen A. Loss-of-function mutations in APOC3 and risk of ischemic vascular disease. *The New England journal of medicine.* Jul 3 2014;371(1):32-41.

31. Khetarpal SA, Zeng X, Millar JS, et al. A human APOC3 missense variant and monoclonal antibody accelerate apoC-III clearance and lower triglyceride-rich lipoprotein levels. *Nature medicine.* Sep 2017;23(9):1086-1094.

32. Klimentidis YC, Chougule A, Arora A, Frazier-Wood AC, Hsu CH. Triglyceride-Increasing Alleles Associated with Protection against Type-2 Diabetes. *PLoS genetics.* May 2015;11(5):e1005204.

33. Fall T, Xie W, Poon W, et al. Using Genetic Variants to Assess the Relationship Between Circulating Lipids and Type 2 Diabetes. *Diabetes.* Jul 2015;64(7):2676-2684.

34. De Silva NM, Freathy RM, Palmer TM, et al. Mendelian randomization studies do not support a role for raised circulating triglyceride levels influencing type 2 diabetes, glucose levels, or insulin resistance. *Diabetes.* Mar 2011;60(3):1008-1018.

35. White J, Swerdlow DI, Preiss D, et al. Association of Lipid Fractions With Risks for Coronary Artery Disease and Diabetes. *JAMA cardiology.* Sep 01 2016;1(6):692-699.

© 2018 Lotta LA et al. *JAMA Cardiol.*
